# Gut microbiota modulation and amino acid absorption by *Lactiplantibacillus plantarum* TWK10 in pea protein ingestion: TWK10 boosts hut microbiota, amino acid uptake

**DOI:** 10.1016/j.crfs.2024.100917

**Published:** 2024-11-09

**Authors:** Mon-Chien Lee, Chun-Hui Chiu, Yi-Chu Liao, Yi-Chen Cheng, Chia-Chia Lee, Chin-Shan Ho, Yi-Ju Hsu, Hsiao-Yun Chang, Jin-Seng Lin, Chi-Chang Huang

**Affiliations:** a Graduate Institute of Sports Science, National Taiwan Sport University, Taoyuan City, 333325, Taiwan; b Center for General Education, Taipei Medical University, Taipei, Taiwan; c Graduate Institute of Health Industry and Technology, Research Center for Food and Cosmetic Safety, College of Human Ecology, Chang Gung University of Science and Technology, Taoyuan, 333324, Taiwan; d Department of Traditional Chinese Medicine, Chang Gung Memorial Hospital, Keelung, 20401, Taiwan; e Culture Collection & Research Institute, SYNBIO TECH INC., Kaohsiung, 82151, Taiwan; f Department of Athletic Training and Health, National Taiwan Sports University, Taoyuan, 333325, Taiwan

**Keywords:** Exercise performance, Muscle strength, Probiotic, Supplement, Branched-chain amino acid*s*

## Abstract

For vegetarians or vegan athletes, improving the utilization of plant-based protein and the absorption of amino acids is crucial. This study explored the impact of combining pea protein with *Lactiplantibacillus plantarum* TWK10 and resistance training on amino acid absorption and exercise performance. Sixteen male and sixteen female participants were randomly assigned to either a control group (20 g of pea protein without TWK10) or a TWK10 group (20 g of pea protein combined with 1 × 10^10^ colony-forming units of TWK10). After 28 days of supplementation combined with resistance exercise training three times per week. All subjects underwent body composition and muscle strength performance, plasma and fecal samples were collected for microbiota analysis and blood amino acid concentrations. The TWK10 group showed a significant increase in muscle thickness and improvements were observed in 1 repetition maximum bench press, explosive, anaerobic power output compared to before the intervention, and were significantly higher than those in the control group (*p* < 0.05). TWK10 supplementation significantly increased the area under the curve and maximum concentration of branched-chain amino acids, essential amino acids, and total amino acids (*p* < 0.05). Furthermore, TWK10 supplementation effectively increased the richness of gut bacterial families. Our study demonstrated that the TWK10 significant increase in the abundance of specific bacterial families in the gut, resulting in increased pea protein amino acid absorption. Moreover, increasing muscle mass and significantly improving muscle thickness, muscle strength, power, and anaerobic capacity.

## Highlights

• Probiotic supplementation boosts amino acid absorption from plant-based protein.
• Probiotic supplementation increases muscle thickness after resistance training.
• Probiotic supplementation enhances muscle strength and explosive power.
• Probiotic supplementation supports intestinal microbiota balance for health.

## Graphical abstract

**Figure:**
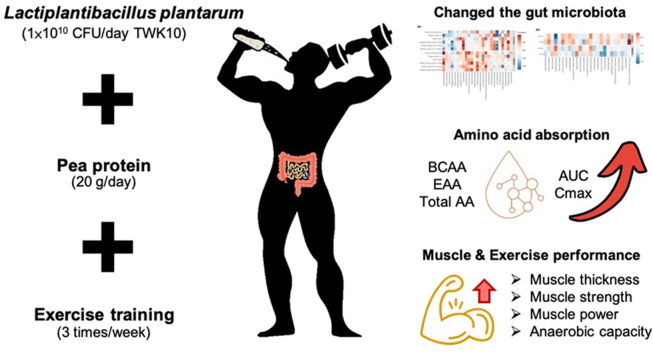

## 1 Introduction

Regular physical activity and a balanced diet are key to maintaining or improving physical function and physique, which in turn reduces the risk of obesity and related comorbidities (Ashaolu et al., 2024). In sports, proper nutritional supplementation strategies play a crucial role in enhancing performance, adapting to training, replenishing energy stores, and minimizing fatigue and recovery time (Kim and Kim, 2020). Among these strategies, protein supplementation has been shown to promote muscle anabolism and, when combined with exercise training, can improve physical performance (Chapman et al., 2021). However, factors such as individual nutritional status, digestive and absorptive capacity, muscle anabolic pathway sensitivity, and the source of protein can influence the anabolic response to protein intake (Trommelen et al., 2023).

Supplementary protein sources are typically classified as either animal-based or plant-based proteins (PBP). Among plant-based options, pea protein is particularly valued for its balanced amino acid profile, lower allergenic potential, and superior absorption (Rathi et al., 2024). Although PBPs provide various health benefits due to their high content of fiber, vitamins, minerals, flavonoids, unsaturated fatty acids, and antioxidants—which have been associated with reduced risks of cardiovascular disease, cancer, and overall mortality (Segovia-Siapco and Sabaté, 2019; Oussalah et al., 2020)—they differ significantly from animal proteins in terms of protein quality and amino acid composition. The reduced anabolic properties of PBPs are often attributed to their lower content of essential amino acids (EAAs) or deficiencies in key amino acids such as leucine, lysine, and methionine (Pinckaers et al., 2021). Therefore, developing effective strategies to enhance the utilization and amino acid absorption of PBPs is critical to addressing current nutritional challenges and mitigating the limitations associated with excessive plant-based dietary intake.

Gut microbes play a vital role in the absorption and metabolism of proteins and amino acids. Probiotics, defined as “live microorganisms” that, when administered in appropriate amounts and strains, confer health benefits to the host (Hill et al., 2014), are considered one of the most effective methods for regulating the intestinal microbiota. Probiotics may improve intestinal permeability and the health of intestinal lining cells by reducing inflammation and promoting the absorption of nutrients, including minerals, peptides, and amino acids (Yan et al., 2024). Probiotic supplementation has been shown to be a promising nutritional strategy for enhancing postprandial blood amino acid levels and addressing deficiencies in plant protein composition (Jäger et al., 2020). A previous animal study demonstrated that supplementation with *Lactiplantibacillus plantarum* could enhance the expression of oligopeptide transporter 1 by modulating protein kinase C activity, resulting in an increased absorption rate of amino acids (Chen et al., 2010).

Our prior research on TWK10, a supplement containing *L. plantarum* isolated from Taiwanese kimchi No. 10, demonstrated improvements in muscle mass and exercise performance (Huang et al., 2019; Lee et al., 2021). However, to date, no studies have demonstrated that *Lactiplantibacillus plantarum* can aid in the absorption and utilization of amino acids following plant-based protein intake. Thus, the aim of this study is to investigate whether *L. plantarum* TWK10 can improve amino acid absorption in humans after consuming pea protein and, when combined with exercise training, enhance muscle mass, strength, and explosive performance. This study will further explore its role through the modulation of the gut microbiota.

## 2 Materials and methods

### 2.1 Participants

This study was conducted in accordance with the Declaration of Helsinki and was approved and reviewed by the Institutional Review Board of Landseed International Hospital (Taoyuan, Taiwan; LSHIRB number 20-037-A2). Sixteen male and sixteen female (N = 32) healthy adult non-athletes aged 20–30 years of age were included. Exclusions were made based on smoking, cardiovascular disease, hypertension, body mass index (BMI) > 27, metabolic disease, asthma or sports injury (nerve, muscle, bone), and relevant stakeholders to the project leader. After a detailed explanation of all the risks and benefits of the experimental procedure, the consenting participants signed the informed consent form in person before starting the experiment. Subjects were required to cooperate by maintaining a normal diet during the experiment and not taking nutritional supplements such as probiotics, or any kind of protein supplements. This trial was registered at clinicaltrials.gov (accessed on June 14, 2022) with the identifier NCT 05412667.The basic information about the participants is presented in Table S1.

### 2.2 Supplementation

The pea protein (Pisane C9) was purchased from Han-Sient Trading Co., Ltd (New Taipei City, Taiwan). Based on a previous study, the daily dose of pea protein supplementation for the subjects was set at 20 g/day (Jäger et al., 2020). *L. plantarum* TWK10 was isolated from Taiwanese traditional kimchi and identified as a subspecies of *L. plantarum* through whole genome sequencing. TWK10 freeze-dried powder, containing 1 × 10^10^ CFU, was produced by SYNBIO TECH INC. (Kaohsiung, Taiwan), and it was mixed with 20 g of pea protein and packed into vacuum bags. The control sample consisted of 20 g of pea protein in the same packaging without TWK10 bacterial powder. TWK10 is one of the strains manufactured using SYNTEK™, which can improve their stability and safety. According to the storage stability test results provided by the manufacturer, cell viability shows almost no decline after storage at the condition of 25 °C/60% RH (Relative Humidity) for 3 months. For long term storage, specifically at 4 °C with ambient humidity for 24 months, the recovery rate is 97%. Therefore, in this study, the participants were instructed to store the supplement in a cool, shaded area at room temperature, and to avoid direct sunlight exposure.

### 2.3 Experimental design

**Fig. 1:**
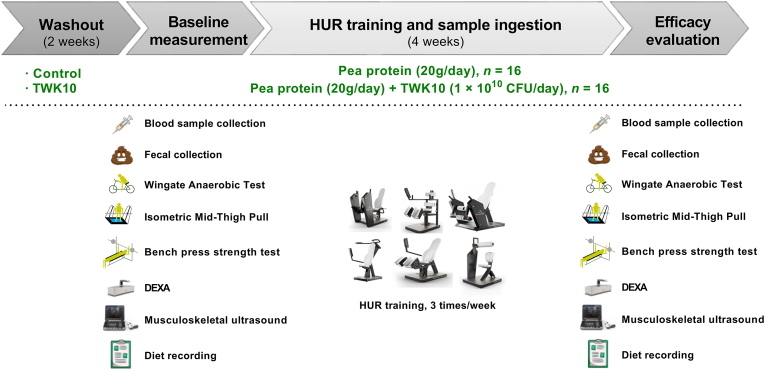
Experimental design.

After all subjects were recruited, numbers were assigned according to the order of registration. Then, when the gender ratio of the two groups was the same, we randomly divided them into two groups through numbered sampling and conducted a double-blind experiment. Sixteen males and 16 females were randomly assigned to two groups (8 male and 8 female participants in each group): the control group (20 g pea protein without TWK10) and the TWK10 group (20 g pea protein combined with 1 × 10^10^ CFU of TWK10). All subjects were instructed to dissolve the sample evenly in 200–250 mL of cold drinking water and take it 2 h before going to bed on rest days, drink it within 30 min after training on training days, and take it before the test on test days for 28 consecutive days. Additionally, they performed resistance training three times a week. After the subjects are admitted, they will first be required to undergo a two-week wash-out period, during which they are prohibited from taking any probiotics, yogurt, kimchi, yogurt and other foods. All subjects underwent tests for body composition, muscle thickness and muscle strength performance at before (baseline measurement) and after the 4 weeks intervention (efficacy evaluation) time points. Stool samples were collected for analysis, and blood samples were collected to analyze changes in blood amino acid concentrations at different time points. Furthermore, all subjects were required to maintain their usual lifestyle and record their dietary intake for 3 days before and after the intervention (2 days on weekdays and 1 day on a weekend) by taking photos of their meals and uploading them to the notepad of the mobile application Line (Z Intermediate Global Corporation, Tokyo, Japan). A nutritionist then analyzed their nutrient intake. The experimental design has shown in Fig. 1, and each icon is described and labeled with its corresponding time point.

### 2.4 Resistance training protocol

All subjects were instructed to perform resistance training three times a week at National Taiwan Sport University gym, using pneumatic resistance training equipment, which included abdominal/back 5310, abduction/adduction 3520-HI5, push-up/pull-down 3120, leg extension/curl 3530, twist rehab 5340, and leg press 5540 (AB Hur Oy, Kokkola, Finland), to target strengthening of the upper limbs, back, abdomen, and legs. Before commencing training, all subjects assessed their 3-RM (repetition maximum) and calculated their 1RM using the coefficient formula from previous literature to determine the appropriate resistance training intensity (Brzycki, 1993). During the first week, they performed a set of 15 repetitions at 60% of 1RM intensity. In the second week, the intensity was increased to 70% of 1RM for a set of 12 repetitions. In the third week, the intensity was further increased to 75% of 1RM for a set of 12 repetitions. Finally, in the fourth week, the subjects maintained 75% of 1RM for 2 sets of 10 repetitions. They were overseen by professional coaches and laboratory staff from the National Taiwan Sport University to ensure all participants used correct training loads and techniques.

### 2.5 Maximal bench press strength performance

We assessed maximal strength performance using the bench press strength test. Following established research methods (Zourdos et al., 2016), participants warmed up to an estimated 80% of their 1RM. After a 5–7 min rest, researchers determined the load for the first 1RM attempt. The load was then increased on each subsequent attempt until reaching 1RM, with 5–7 min of rest between each attempt. During the test, participants were allowed to choose their grip distance, but it had to remain consistent during the front and rear tests. Weightlifting belts, wrist guards, elbow pads, chalk, and other weightlifting equipment were not permitted. A repetition was counted only if the participant fully lowered the barbell to the chest and pushed it up until the arms were fully extended.

### 2.6 Isometric Mid-thigh pull (IMTP)

*As previously described by*Lee et al. (2022), we utilized a force plate (Model 9260AA, Kistler, Winterthur, Switzerland) and custom-made IMTP test software (IMTP Rack, Kairos Strength, USA) to measure the maximum force generated by all subjects. Participants were instructed to stand with their feet shoulder-width apart, barbell between their thighs, torso erect, spine neutral, knees and hips at a 140° angle, and pull the barbell with maximum force for 5 s. Before testing, subjects underwent familiarization with the IMTP testing method and exercises to ensure proper form and stable weight frequency. Measurements were repeated at 2-min intervals for each test to minimize detection errors associated with posture changes.

### 2.7 Wingate anaerobic test (WAnT)

We evaluated the subjects' strength performance during a full sprint lasting 30 s using an anaerobic power bicycle (894E, Monark Exercise AB, Vansbro, Sweden). The seat height could be adjusted for each participant, and tensioning toe clips were used to keep the feet from slipping off the pedals. The subjects sprinted at 120 rpm, and an additional resistance equivalent to 7.5% of their body weight was set (e.g., an 80 kg person would add 6 kg of resistance). The sprinting and timing started simultaneously and continued for 30 s. We followed the descriptions from previous research (Lee et al., 2021).

### 2.8 Body composition

Whole-body composition measurements were conducted using a noninvasive dual-energy X-ray absorptiometry laboratory (Lunar iDXA, GE Healthcare, Chicago, IL, USA). DXA was calibrated according to manufacturer's protocol prior to measurement. All subjects should complete the DXA test before noon, and the pretest and posttest need to be conducted at the same time. Subjects were asked to fast 12 h before the test and not drink liquids 2 h before the test. The subjects were required to lie flat on the test bed, with their body aligned along the center line and limbs within the detection range. Two types of X-rays with different energies were used to scan the inspected parts, and the scintillation detector received the X-rays that penetrated the inspected parts. This allowed for the calculation and analysis of parameters such as muscle mass and body fat.

### 2.9 Ultrasonic measurement of muscle and fascia thickness

Muscle and fascia thickness of the biceps brachii and quadriceps femoris muscles were measured using an ultrasonic scanner (H1300, BENQ, Taipei, Taiwan). The participant was asked to lie flat on their back with their palms facing up, assuming an anatomical position. For the biceps brachii, the measurement position was the straight-line distance from the acromion to the transverse crease of the elbow, with the ultrasonic probe placed at 1/3 of the transverse crease of the elbow. For the quadriceps femoris, the muscle probe was placed from the anterior superior iliac spine (ASIS) to the patella at a 1/2 straight-line distance from the edge (Mah and van Alfen, 2018) Muscle thickness analysis involved dividing the sum of the thickness of the two ends of the muscle by two. The muscle fascia calculation method was based on the calculation method of muscle fascia (Nakamura et al., 2011), which is: muscle thickness × Sin(θ), where θ is the angle between muscle fascia and the horizontal line. ImageJ version 2.3.0/1.53f (National Institutes of Health, Maryland, USA) was used for analysis.

### 2.10 Amino acid absorption analysis

Blood was drawn from all subject's veins in the antecubital fossa at baseline, 30 min, 60 min, 120 min, and 180 min post ingestion. The plasma was collected from all blood samples by centrifugation at 1500×*g* for 15 min at 4 °C. The Kairos amino acid kit (Waters, Milford, MA, USA) were used to quantify amino acids in human plasma according to the manufacturer's protocol. The LC-MS/MS was used for analysis. The CORTESTM UPLC C18 (1.6 μm, 2.1 × 150 mm) column was employed and maintained at 55 °C with a flow rate at 0.5 mL/min and injected 1.5 μL. The mobile phase consisted of water (A) and acetonitrile (B), both containing 0.1% formic acid. The linear gradient conditions were: 1%B (0–1 min), 1–13%B (1–2 min), 13–15%B (2–5.5 min), 15–95%B (5.5–6.5 min), 95%B (6.5–7.5 min). All data for analytes were collected by multiple reaction monitoring (MRM) mode and MassLynx4.1 software.

The optimized MRM conditions are shown in Table S2. Most amino acids were analyzed in positive mode, and the collision energy for most amino acids was 60eV. The calibration curves ranged from 5 to 4000 μM, while for good intensity amino acids, the range was 5–1000 μM. Fig. S1 demonstrated the efficient separation of the 23 amino acids in 7.5 min and showed linearity with a coefficient of correlation (*r*) greater than 0.9950 (Table S3).

### 2.11 DNA extraction

Fresh stoolsamples were suspended in phosphate-buffered saline (PBS) at a ratio of 1:9 and vortexed until a homogeneous suspension was obtained. A 200 μL homogenized suspension was then washed twice with 1 mL of PBS and centrifuged at 16,200×*g* for 5 min, and 1 mL of the supernatant was discarded. Fecal DNA was subsequently extracted using a modified method developed by Zhu et al. (1993). To the 200 μL washed fecal suspension, 700 mg of glass beads (product No. 11079101, 0.1 mm; BioSpec Products, Bartlesville, OK, USA), 250 μL of extraction buffer (containing 100 mM Tris-HCl, 40 mM EDTA, pH 9.0), and 500 μL of the phenol-chloroform isoamyl alcohol mixture (product No. 77617, Sigma-Aldrich) were added. The mixture was then homogenized at 6.5 m/s for 30 s using FastPrep-24™ (MP Biomedicals, USA). After homogenization, the mixture was added to 50 μL of 10% SDS and heated at 50 °C for 20 min. Next, it was mixed with 150 μL of 3 M sodium acetate on ice for 5 min and centrifuged at 16,200×*g* for 5 min at 4 °C. The supernatant was transferred to a new 1.5 mL centrifuge tube and mixed with 450 μL of isopropanol to precipitate DNA, followed by centrifugation at 16,200 g for 10 min at 4 °C. The DNA pellet was washed twice in 70% ethanol and centrifuged at 16,200×*g* for 1 min at 4 °C. After centrifugation, the supernatant was discarded, and the DNA pellet was heated at 65 °C for 10–15 min. The dried DNA pellet was then resuspended in 30 μL of ddH_2_O and stored at −20 °C until further analysis.

### 2.12 16S rRNA gene sequencing and bioinformatics processing

The V3-V4 region of the 16S rRNA gene was amplified using specific primers (341F: 5′-CCTACGGGNGGCWGCAG-3′ and 805R: 5′-GACTACHVGGGTATCTAATCC-3′) following the 16S Metagenomic Sequencing Library Preparation procedure (Illumina). Amplicon pools were sequenced on the Illumina MiSeq™ sequencing platform (Illumina, San Diego, CA, USA). Bioinformatic analysis was conducted with QIIME2 as previously described (Bolyen et al., 2019). Based on the characteristics of the compositional data, networks of specific families in each group were built using SparCC correlation coefficients (28. Friedman and Alm, 2012). The networks were visualized using Cytoscape (version 3.8.2; https://github.com/cytoscape/cytoscape/releases/3.8.2/, accessed on November 23, 2021). Correlations between the relative abundances of taxa and exercise performance indices and total amino acids were performed using Spearman correlation. The Kyoto Encyclopedia of Genes and Genomes (KEGG; https://www.genome.jp/kegg/, accessed on December 6, 2021) database was used to analyze pathway enrichment, utilizing Phylogenetic Investigation of Communities by Reconstruction of Unobserved States (PICRUSt2). The raw sequence files supporting the findings of this article are deposited in the NCBI Sequence Read Archive (SRA) database, with the project accession number PRJNA953842.

### 2.13 Statistical analysis

#### 2.13.1 Statistical analysis between and within groups

Data are expressed as mean ± SD. Analysis was performed using the statistical software package SPSS Statistics 25 (IBM Co., Armonk, NY, USA). For comparisons before and after intervention within the group, paired Student's *t*-test were used for parametric analysis, and for non-parametric data, the Wilcoxon signed-rank test was used. For between-group comparisons, unpaired Student's *t*-test was used for parametric analyses, and non-parametric data were analyzed using the Mann-Whitney *U* test. A *p*-value <0.05 indicated a significant difference. The two-way repeated measures ANOVA to measured group, time and group × time effect.

#### 2.13.2 Amino acid concentration AUC, Cmax and Tmax calculation

Cmax is defined as the maximum observed concentration, and time to reach Cmax (Tmax) is the time to reach Cmax. The AUC of the concentrations of the 23 amino acids, as well as BCAA, EAA, and total amino acids, was calculated using the linear trapezoidal rule with all available time points.

#### 2.13.3 Gut microbiota data analysis

Differences in alpha diversities (Observed ASVs and Shannon index) were tested by the Wilcoxon signed-rank test in R. Beta diversity analysis was performed using non-metric multidimensional scaling (NMDS) plots based on Bray-Curtis distances, and visualized using ggplot2 (version 3.4.1) and ggpubr (version 0.6.0). Permutational multivariate analysis of variance (PERMANOVA)/Adonis tests were conducted using vegan: Community Ecology Package (version 2.6.4). To test the effect of different factors on community structure, the envfit function of the vegan package was used with 999 permutations. Differential abundance analysis of taxa was performed using ANCOMBC (Analysis of Compositions of Microbiomes with Bias Correction) methodology (version 1.6.4). P-values were adjusted with the Benjamini & Hochberg method using a cut-off of 0.05. Principal Coordinate Analysis (PCoA) was performed to depict sample dispersions and variations based on KEGG orthologous groups (KOs) and KEGG pathway profiles. PERMANOVA and PCoA were both conducted using the Bray-Curtis dissimilarity matrix. Downstream differential analysis of KEGG pathways was conducted in LEfSe (linear discriminant analysis [LDA] effect size) with default parameters (The threshold for the LDA score was 2.0, and P-values were adjusted with the Bonferroni method). Spearman's correlation coefficient was used for analyses of correlations between gut microbial abundances and exercise-associated phenotypic features.

## 3 Results

Effects on Dietary Intake and Body Composition Before and After 4 Weeks of Pea Protein Combined with TWK10 Administration.

There were no significant differences in daily dietary intake (carbohydrate, protein, and fat) and total calories between the groups. (Table S4).

Similarly, there were no significant differences in body weight, BMI, lean body mass (LBM), or fat mass (FM) between the groups before and after the 4 weeks of administration. However, There was a significant time effect on LBM (*p* = 0.002) and FB (*p* = 0.021), and significant group × time interaction effect on body weight (*p* = 0.002) and BMI (*p* < 0.001) (Table S5).

### 3.1 Effect of supplementation with pea protein combined with TWK10 on muscle and fascia thickness

**Fig. 2:**
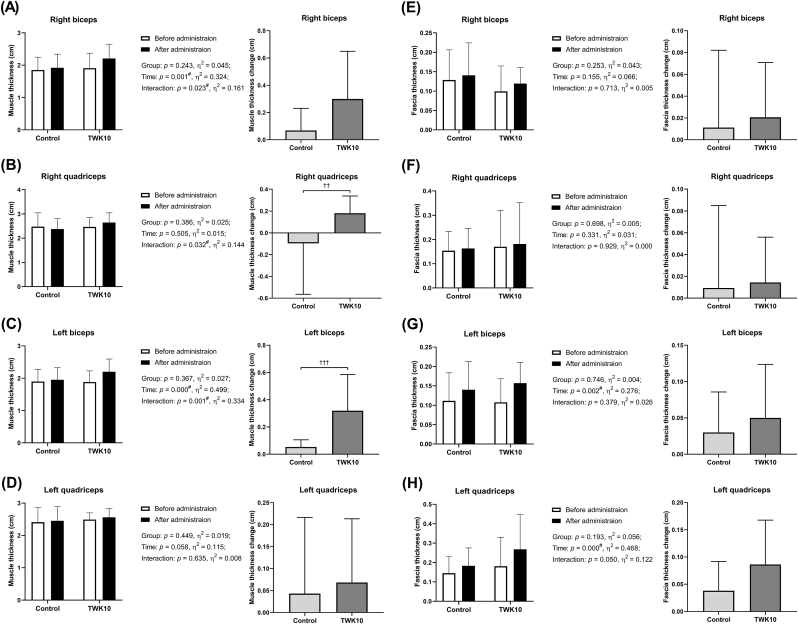
Effects of TWK10 administration on (A) right biceps muscle thickness and change, (B) right quadriceps muscle thickness and change, (C) left biceps muscle thickness and change, (D) left quadriceps muscle thickness and change, (E) right biceps fascia thickness and change, (F) right quadriceps fascia thickness and change, (G) left biceps fascia thickness and change, and (H) left quadriceps fascia thickness and change. Data are shown as mean ± SD. Treatment effect was analyzed by unpaired Student's t-test. ^#^ presented a significant effect by repeated measures ANOVA (*p* < 0.05). The changes in muscle and fascia thickness were calculated as the difference between after and before administration, and statistical significance was analyzed using the Mann-Whitney *U* test. †*p* < 0.05.

As shown in Fig. 2A–D, there were no significant differences in muscle thickness between the control group and the TWK10 group, either before or after the administration. However, in the TWK10 group, the increase in muscle thickness of the right biceps 1.16-fold (*p* = 0.001) and left biceps 1.17-fold (*p* = 0.001) had a significant time effect, and there was a significant interaction between the increase in the right quadriceps right quadriceps by 1.07-fold (*p* < 0.001) and left and left biceps muscles (*p* < 0.001). Additionally, when comparing the delta values before and after, the TWK10 group showed a significant increase in muscle thickness of the right quadriceps (*p* = 0.006) and left biceps (*p* < 0.001) compared to the control group.

Furthermore, in the present study, in addition to the observed increase in muscle thickness, we found that although not significantly different from the control group, 4 weeks of pea protein combined with TWK10 supplementation increased the fascia thickness of the left biceps by 1.46-fold (*p* = 0.002) and the left quadriceps by 1.48-fold (*p* < 0.001), had a significant time effect, respectively. (Fig. 2E–H).

### 3.2 Effect of supplementation with pea protein combined with TWK10 on 1-RM bench press test

**Fig. 3:**
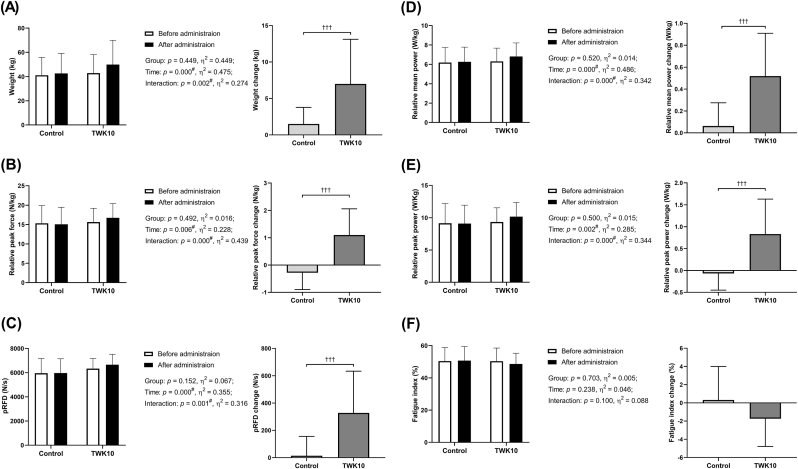
Effects of TWK10 administration on exercise and strength performance (A) 1-RM bench press test and change (B) IMTP test of peak force and change, (C) IMTP test pRFD and change, (D) Wingate test of relative mean power and change, (E) Wingate test of relative peak power and change, and (F) Wingate test of fatigue index and change. Data are shown as mean ± SD. Treatment effects on Relative mean power, Relative peak power, and Fatigue index were analyzed by Mann-Whitney *U* test. ^#^ presented a significant effect by repeated measures ANOVA (*p* < 0.05). The changes in Relative mean power, Relative peak power, and Fatigue index were analyzed using the Mann-Whitney *U* test. ^†^*p* < 0.05.

There were no significant differences in the 1-RM bench press test between the control group and the TWK10 group, either before or after the administration. However, after 4 weeks of supplementation, the TWK10 group improvements in the 1-RM bench press test by 1.04-fold, and had a significantly time effect (*p* < 0.001) and interaction effect (*p* = 0.002). Furthermore, the delta value of the 1-RM bench press muscle strength before and after administration in the TWK10 group was significantly increased compared to the control group (*p* < 0.001) (Fig. 3A).

### 3.3 Effect of supplementation with pea protein combined with TWK10 on IMTP test

Although there were no significant differences between the control group and the TWK10 group in terms of relative peak force and peak RFD (Rate of Force Development) in the IMTP test before and after administration, after 4 weeks of supplementation with TWK10, there were significant improvements of 1.07-fold in relative peak force, and had a significant time effect (*p* = 0.006) and group × time interactions effect (*p* < 0.001). Also increased 1.05-fold in peak RFD and had a significant time effect (*p* < 0.001) and group × time interactions effect (*p* = 0.001). Additionally, the delta value of relative peak force and peak RFD before and after administration in the TWK10 group was significantly higher than in the control group (*p* < 0.001) (Fig. 3B and C).

### 3.4 Effect of supplementation with pea protein combined with TWK10 on anaerobic wingate test

Similarly, there were no significant differences between the control group and the TWK10 group in terms of relative mean power, relative peak power, and fatigue index in the Wingate test before and after administration. However, after 4 weeks of supplementation with TWK10, there were improvements of 1.08-fold in relative mean power, 1.09-fold in relative peak power, and had a significant time effect (*p* < 0.001), (*p* < 0.001) and group × time interactions effect (*p* = 0.002), (*p* < 0.001), respectively, compared to before administration. Moreover, the delta value of relative mean power and relative peak power before and after administration in the TWK10 group was significantly higher than in the control group (*p* < 0.001) (Fig. 3D–F).

### 3.5 Effect of supplementation with pea protein combined with TWK10 on amino acid absorption rate

Overall, regardless of supplementation with pea protein or pea protein combined with TWK10, when combined with 4 weeks of exercise training, the concentrations of most plasma amino acids’ AUC were increased (*p* < 0.05). However, TWK10 supplementation increased the area under the curve of total BCAA by 1.08-fold, had a significant time effect (*p* < 0.001) and interaction effect (*p* < 0.001). Additionally, a clearer picture of the change in the AUC of each amino acid concentration in the plasma before and after supplementation could be seen through the delta value. The results showed that, compared to the control group, TWK10 supplementation significantly reduced the delta AUC of taurine, phenylalanine, and tryptophan (*p* < 0.05), but significantly increased the delta AUC of alanine, alpha-aminobutyric acid, cystine, L-ornithine, glutamine, glycine, tyrosine, leucine, isoleucine, valine, total BCAA, total EAA, and total amino acids (*p* < 0.05) (Table 1).

**Table 1:** Effect of 4 weeks pea protein combined with TWK10 administration on individual amino acids, BCAA, EAA, and total amino acid AUC.

Amino acid AUC (μmol/L•180 min)	Control									TWK10									ANOVA					
	Before			After			Change			Before			After			Change			Group		Time		Interaction	
																			*p*	η^2^	*p*	η^2^	*p*	η^2^
**Alanine**	81810.9	±	14608.2	86432.5	±	10976.3	4621.6	±	3938.3	79209.7	±	17026.9	80676.7	±	13034.3	1467.0	±	4617.4^†^	0.403	0.023	<0.001^#^	0.349	0.046^#^	0.126
**Alpha Aminobutyric Acid**	4367.9	±	869.9	4102.0	±	1102.7	−265.9	±	335.2	4722.3	±	1790.6	4900.0	±	1621.8	177.8	±	973.9^†^	0.236	0.046	0.734	0.004	0.095	0.090
**Arginine**	27689.4	±	6724.7	28672.4	±	5099.1	983.0	±	1943.3	26355.4	±	5503.6	27970.6	±	5882.9	1615.2	±	958.0	0.622	0.008	<0.001^#^	0.434	0.252	0.043
**Asparagine**	16108.3	±	2869.3	17096.5	±	3323.8	988.2	±	547.6	16714.5	±	2301.9	17552.5	±	3111.9	838.0	±	935.3	0.608	0.009	<0.001^#^	0.602	0.583	0.010
**Aspartic Acid**	3442.1	±	1043.9	3736.7	±	1478.8	294.6	±	486.1	3387.8	±	1011.5	3635.2	±	1100.1	247.3	±	292.7	0.850	0.001	0.001^#^	0.327	0.741	0.004
**Citrulline**	5797.1	±	989.7	5919.5	±	882.4	122.3	±	314.6	5861.1	±	1713.0	6134.2	±	1333.6	273.1	±	491.1	0.756	0.003	0.011^#^	0.197	0.309	0.034
**Cystine**	3403.6	±	447.8	5163.2	±	751.8	1759.6	±	334.0	3139.1	±	619.7	5157.8	±	830.7	2018.7	±	284.2^†^	0.568	0.011	<0.001^#^	0.975	0.025^#^	0.157
**L-Ornithine**	21460.5	±	4996.6	20702.6	±	4017.8	−757.9	±	1954.6	17861.4	±	4114.0∗	20978.8	±	4836.3	3117.4	±	1118.9^†^	0.298	0.036	<0.001^#^	0.369	<0.001^#^	0.612
**Glutamine**	153670.8	±	22643.0	151886.6	±	20908.4	−1784.2	±	6056.1	147362.4	±	18267.2	157165.0	±	19856.7	9802.6	±	2884.5^†^	0.943	<0.001	<0.001^#^	0.432	<0.001^#^	0.614
**Glycine**	54315.0	±	10099.1	52308.8	±	9035.0	−2006.2	±	2238.5	52225.2	±	11125.4	53149.0	±	9622.9	923.8	±	2068.0^†^	0.860	0.001	0.166	0.063	0.001^#^	0.330
**Proline**	53356.9	±	15871.7	58457.7	±	13017.5	5100.8	±	4104.9	48139.0	±	12686.5	51338.9	±	13515.5	3199.9	±	4168.3	0.212	0.051	<0.001^#^	0.518	0.204	0.053
**Serine**	29219.6	±	4430.3	30839.7	±	4849.5	1620.1	±	781.3	29523.7	±	3632.4	30749.6	±	6548.0	1226.0	±	3072.6	0.951	<0.001	0.001^#^	0.301	0.622	0.008
**Taurine**	15922.8	±	3305.6	15051.0	±	3621.6	−871.8	±	486.6	17970.8	±	3605.7	13157.8	±	3997.4	−4813.0	±	820.1^†^	0.952	<0.001	<0.001^#^	0.950	<0.001^#^	0.901
**Tyrosine**	16930.8	±	2850.7	17866.7	±	2032.3	935.9	±	881.4	16537.3	±	2397.3	16683.9	±	2841.0	146.6	±	923.6^†^	0.382	0.026	0.002^#^	0.277	0.019^#^	0.169
**Leucine**	37532.3	±	5972.6	37382.7	±	5597.3	−149.6	±	1015.2	35079.5	±	5837.7	37512.7	±	5668.1	2433.2	±	809.0^†^	0.572	0.011	<0.001^#^	0.623	<0.001^#^	0.679
**Isoleucine**	18020.7	±	2791.9	17010.8	±	3174.7	−1010.0	±	656.7	17598.8	±	3472.8	19331.7	±	6419.3	1733.0	±	3790.4^†^	0.506	0.015	0.458	0.018	0.008^#^	0.213
**Valine**	59547.4	±	10092.8	59240.4	±	7664.1	−306.9	±	3623.6	52281.7	±	8357.7∗	56601.9	±	7863.4	4320.3	±	2649.7^†^	0.106	0.085	0.001^#^	0.299	<0.001^#^	0.362
**Histidine**	18222.3	±	2509.4	19107.9	±	2012.4	885.7	±	587.5	18729.9	±	2001.1	19730.8	±	23.91.5	1000.9	±	490.7	0.478	0.017	<0.001^#^	0.764	0.552	0.012
**Lysine**	49994.9	±	7762.0	52165.4	±	7362.3	2170.5	±	1857.9	48895.1	±	5992.9	50590.8	±	7385.1	1695.7	±	2050.6	0.598	0.009	<0.001^#^	0.510	0.498	0.015
**Methionine**	5386.5	±	956.9	5640.6	±	1164.1	254.1	±	390.0	5139.3	±	658.8	5618.2	±	1395.7	478.9	±	774.6	0.715	0.005	0.002^#^	0.276	0.308	0.035
**Phenylalanine**	15561.2	±	2098.4	16233.9	±	1955.2	672.8	±	656.3	16252.0	±	1848.0	16036.4	±	1892.1	−215.6	±	378.6^†^	0.721	0.004	0.022^#^	0.163	<0.001^#^	0.423
**Threonine**	31541.6	±	5526.5	31902.6	±	6428.4	360.9	±	1281.2	31112.2	±	6167.8	31402.8	±	8956.1	290.6	±	3286.5	0.848	0.001	0.466	0.018	0.937	<0.001
**Tryptophan**	20593.8	±	3343.4	22210.8	±	4553.1	1617.0	±	1792.4	21234.1	±	3190.9	20945.4	±	3303.2	−288.7	±	937.6^†^	0.806	0.002	0.013^#^	0.187	0.001^#^	0.321
**BCAA**	115100.4	±	16049.6	113633.9	±	13694.4	−1466.5	±	3726.3	104959.9	±	12899.2	113446.3	±	13316.3	8486.4	±	4199.7^†^	0.302	0.035	<0.001^#^	0.455	<0.001^#^	0.626
**EAA**	256400.7	±	22798.1	260895.1	±	21445.8	4494.4	±	4811.5	246322.5	±	16784.2	257770.7	±	20788.9	11448.2	±	8671.7^†^	0.364	0.027	<0.001^#^	0.580	0.009^#^	0.208
**Total amino acid**	743896.5	±	60680.5	759131.2	±	53011.6	15234.7	±	12883.8	715332.3	±	54404.8	747020.8	±	60978.6	31688.5	±	11382.9^†^	0.321	0.033	<0.001^#^	0.799	0.001^#^	0.328

Data are shown as mean ± SD. Treatment effect was analyzed by unpaired Student's *t*-test. ∗*p* < 0.05. ^#^ presented a significant effect by repeated measures ANOVA (*p* < 0.05). The changes on AUC were analyzed using the Mann-Whitney *U* test. ^†^*p* < 0.05.

Furthermore, administration of TWK10 had a significant time effect (*p* < 0.05) and group × time interactions effect (*p* < 0.05) on increased the Cmax of most amino acids, total BCAA, total EAA, and total amino acids, except for taurine, tyrosine, and phenylalanine, which showed lower Cmax values (Table 2). In contrast, the control group had lower Cmax values for most amino acids than before the administration. Similarly, by comparing the delta values, the TWK10 group had significantly higher delta Cmax values for alanine, cystine, L-ornithine, glutamine, glycine, serine, leucine, isoleucine, valine, histidine, methionine, total BCAA, total EAA, and total amino acids compared to the control group (*p* < 0.05) (Table 2). However, supplementation with TWK10 significantly reduced the delta Cmax values of taurine, phenylalanine, and tryptophan compared to the control group (*p* < 0.05).

**Table 2:** Effect of 4 weeks pea protein combined with TWK10 administration on individual amino acids, BCAA, EAA, and total amino acid Cmax.

Amin acid Cmax (μmol/L)	Control									TWK10									ANOVA					
	Before			After			Change			Before			After			Change			Group		Time		Interaction	
																			*p*	η^2^	*p*	η^2^	*p*	η^2^
**Alanine**	526.6	±	74.9	526.0	±	81.2	−0.6	±	17.1	483.1	±	116.3	497.8	±	80.1	14.7	±	47.1^†^	0.782	0.003	0.032^#^	0.145	0.117	0.080
**Alpha Aminobutyric Acid**	26.3	±	5.5	25.3	±	6.8	−1.0	±	1.8	28.5	±	10.6	29.3	±	9.6	0.8	±	5.7	0.718	0.004	0.994	<0.001	0.540	0.013
**Arginine**	195.2	±	54.4	196.0	±	37.5	0.8	±	25.9	177.7	±	37.5	189.1	±	53.6	11.3	±	17.5	0.894	0.001	<0.001^#^	0.981	0.001^#^	0.329
**Asparagine**	110.1	±	22.1	116.8	±	27.2	6.7	±	6.7	112.0	±	16.6	118.5	±	26.8	6.5	±	12.7	0.456	0.019	0.005^#^	0.237	<0.001^#^	0.493
**Aspartic Acid**	24.6	±	7.2	25.0	±	9.5	0.4	±	3.4	23.0	±	7.8	25.1	±	7.4	2.2	±	2.9	0.783	0.003	0.046^#^	0.127	<0.001^#^	0.535
**Citrulline**	35.4	±	5.6	35.2	±	5.2	−0.3	±	1.8	36.1	±	10.3	36.4	±	7.9	0.3	±	2.9	0.704	0.005	0.037^#^	0.137	<0.001^#^	0.380
**Cystine**	20.7	±	2.0	38.2	±	4.7	17.5	±	2.8	18.7	±	3.7	39.9	±	6.1	21.2	±	2.7^†^	0.152	0.067	0.897	0.001	0.628	0.008
**L-Ornithine**	132.8	±	34.9	126.5	±	23.9	−6.3	±	16.6	110.3	±	27.5	132.8	±	36.8	22.5	±	13.3^†^	0.860	0.001	0.056	0.116	0.500	0.015
**Glutamine**	937.5	±	115.5	889.7	±	132.4	−47.8	±	44.8	891.1	±	98.9	913.9	±	107.6	22.8	±	17.3^†^	0.694	0.005	<0.001^#^	0.832	<0.001^#^	0.735
**Glycine**	337.3	±	60.1	312.8	±	51.6	−24.5	±	16.0	313.1	±	72.3	321.1	±	53.3	8.0	±	25.7^†^	0.290	0.037	0.153	0.067	0.086	0.095
**Proline**	366.4	±	99.9	368.4	±	81.3	1.9	±	28.4	324.8	±	80.8	321.5	±	82.4	−3.3	±	32.3	0.763	0.003	0.873	0.001	<0.001^#^	0.551
**Serine**	185.9	±	28.6	194.0	±	30.1	8.1	±	8.0	186.0	±	24.6	190.0	±	43.2	4.0	±	23.0^†^	0.632	0.008	0.089	0.094	0.028^#^	0.150
**Taurine**	101.9	±	19.1	98.1	±	19.9	−3.8	±	7.2	110.3	±	19.8	83.9	±	23.3	−26.4	±	6.8^†^	0.180	0.059	0.391	0.025	0.006^#^	0.227
**Tyrosine**	111.4	±	17.3	111.7	±	13.4	0.3	±	5.9	107.4	±	14.6	104.1	±	16.7	−3.3	±	5.6	0.561	0.011	<0.001^#^	0.873	<0.001^#^	0.351
**Leucine**	258.5	±	44.0	248.8	±	42.8	−9.7	±	9.8	244.8	±	35.8	254.0	±	34.8	9.2	±	7.7^†^	0.578	0.010	0.331	0.032	0.712	0.005
**Isoleucine**	126.4	±	18.2	122.6	±	22.6	−3.8	±	8.4	116.3	±	27.7	144.8	±	71.0	28.5	±	55.5^†^	0.686	0.006	<0.001^#^	0.375	0.137	0.072
**Valine**	385.5	±	75.2	365.9	±	44.6	−19.5	±	35.4	344.3	±	49.8	355.0	±	47.4	10.7	±	20.1^†^	0.741	0.004	<0.001^#^	0.371	0.042^#^	0.131
**Histidine**	114.3	±	13.2	121.3	±	10.7	7.0	±	4.2	114.2	±	12.3	126.7	±	14.9	12.5	±	3.5^†^	0.725	0.004	0.447	0.019	0.559	0.012
**Lysine**	339.2	±	45.6	347.0	±	50.7	7.8	±	26.8	331.3	±	44.0	334.8	±	67.8	3.5	±	36.8	0.760	0.003	0.016^#^	0.178	<0.001^#^	0.375
**Methionine**	34.3	±	4.4	36.3	±	7.8	2.0	±	4.3	32.3	±	4.3	36.5	±	8.0	4.2	±	3.9^†^	0.473	0.017	0.323	0.033	<0.001^#^	0.487
**Phenylalanine**	101.8	±	11.3	100.4	±	12.0	−1.5	±	4.8	104.7	±	12.1	100.2	±	10.0	−4.5	±	3.1^†^	0.419	0.022	0.050^#^	0.122	0.004^#^	0.243
**Threonine**	199.3	±	36.2	194.9	±	39.3	−4.4	±	11.2	192.2	±	36.2	191.6	±	55.1	−0.6	±	23.4	0.196	0.055	0.010^#^	0.199	<0.001^#^	0.610
**Tryptophan**	129.1	±	17.2	138.7	±	23.5	9.6	±	9.3	133.0	±	17.6	130.6	±	18.6	−2.4	±	6.5^†^	0.379	0.026	<0.001^#^	0.969	0.372	0.027
**BCAA**	770.3	±	116.2	737.3	±	92.7	−33.1	±	31.7	705.4	±	78.6	753.8	±	95.2	48.4	±	52.1^†^	0.958	<0.001	<0.001^#^	0.987	0.980	<0.001
**EAA**	1688.4	±	152.2	1675.8	±	134.7	−12.6	±	46.9	1613.2	±	106.9	1674.2	±	148.3	61.1	±	82.7^†^	0.673	0.006	<0.001^#^	0.994	0.684	0.006
**Total amino acid**	4800.6	±	352.5	4739.5	±	334.1	−61.1	±	85.0	4535.2	±	341.7∗	4677.5	±	380.4	142.2	±	82.9^†^	0.547	0.012	<0.001^#^	0.995	0.560	0.011

Data are shown as mean ± SD. Treatment effect was analyzed by unpaired Student's *t*-test. ∗*p* < 0.05. ^#^ presented a significant effect by repeated measures ANOVA (*p* < 0.05). The changes on Cmax were analyzed using the Mann-Whitney *U* test. ^†^*p* < 0.05.

The control or TWK10 groups were decreased time to maximum concentration (Tmax) of monomeric amino acids for total EAA and total amino acids, had a significant time effect (*p* < 0.001), while there was no significant difference in Tmax for BCAA. Furthermore, when comparing the delta values, supplementation with TWK10 resulted in a significant decrease in Tmax for aspartic acid and lysine (*p* < 0.05), but a significant increase in Tmax for citrulline, glycine, taurine, isoleucine, and total BCAA compared to the control group, respectively (*p* < 0.05) (Table 3).

**Table 3:** The effect of 4 weeks of pea protein combined with TWK10 administration on individual amino acids, BCAA, EAA, and total amino acid time to maximum concentration (Tmax).

Amin acid Tmax (min)	Control									TWK10									ANOVA					
	Before			After			Change			Before			After			Change			Group		Time		Interaction	
																			*p*	η^2^	*p*	η^2^	*p*	η^2^
**Alanine**	63.8	±	24.2	71.3	±	42.2	7.5	±	52.0	54.4	±	12.1	69.4	±	32.3	15.0	±	39.5	0.407	0.023	0.178	0.060	0.649	0.007
**Alpha Aminobutyric Acid**	45.0	±	15.5	33.8	±	10.2	−11.3	±	21.6	45.0	±	24.5	37.5	±	30.0	−7.5	±	40.2	0.711	0.005	0.111	0.083	0.745	0.004
**Arginine**	39.4	±	14.4	35.6	±	12.1	−3.8	±	15.0	37.5	±	23.2	41.3	±	15.0	3.8	±	28.7	0.666	0.006	1.000	0.000	0.362	0.028
**Asparagine**	56.3	±	10.2	35.6	±	12.1	−20.6	±	14.4	48.8	±	15.0	33.8	±	10.2	−15.0	±	15.5	0.171	0.061	<0.001^#^	0.603	0.295	0.036
**Aspartic Acid**	43.1	±	15.4	65.6	±	22.5	22.5	±	30.0	82.5	±	30.0∗	35.6	±	12.1∗	−46.9	±	32.8^†^	0.356	0.028	0.036^#^	0.138	<0.001^#^	0.565
**Citrulline**	52.5	±	13.4	63.8	±	15.0	11.3	±	24.2	39.4	±	14.4∗	71.3	±	30.7	31.9	±	20.4^†^	0.628	0.008	<0.001^#^	0.498	0.014^#^	0.185
**Cystine**	63.8	±	15.0	0.0	±	0.0	−63.8	±	15.0	67.5	±	27.9	0.0	±	0.0	−67.5	±	27.9	0.640	0.007	<0.001^#^	0.901	0.640	0.007
**L-Ornithine**	61.9	±	25.6	67.5	±	20.5	5.6	±	36.7	63.8	±	24.2	71.3	±	30.7	7.5	±	40.2	0.638	0.007	0.343	0.030	0.891	0.001
**Glutamine**	46.9	±	15.4	71.3	±	24.2	24.4	±	27.3	35.6	±	12.1∗	73.1	±	28.9	37.5	±	33.8	0.373	0.027	<0.001^#^	0.520	0.236	0.046
**Glycine**	56.3	±	10.2	46.9	±	37.9	−9.4	±	39.1	35.6	±	16.3∗	43.1	±	15.4	7.5	±	17.3^†^	0.049^#^	0.123	0.862	0.001	0.125	0.077
**Proline**	30.0	±	0.0	46.9	±	15.4	16.9	±	15.4	33.8	±	10.2	41.3	±	15.0	7.5	±	20.5	0.733	0.004	0.001^#^	0.326	0.154	0.067
**Serine**	52.5	±	13.4	45.0	±	26.8	−7.5	±	33.8	58.1	±	7.5	41.3	±	15.0	−16.9	±	15.4	0.813	0.002	0.013^#^	0.187	0.320	0.033
**Taurine**	63.8	±	15.0	116.3	±	15.0	52.5	±	20.5	84.4	±	33.3∗	99.4	±	32.3	15.0	±	47.7^†^	0.766	0.003	<0.001^#^	0.474	0.007^#^	0.217
**Tyrosine**	63.8	±	15.0	58.1	±	7.5	−5.6	±	16.3	60.0	±	0.0	54.4	±	12.1	−5.6	±	12.1	0.164	0.063	0.034^#^	0.141	1.000	<0.001
**Leucine**	60.0	±	0.0	39.4	±	14.4	−20.6	±	14.4	63.8	±	15.0	45.0	±	15.5	−18.8	±	18.6	0.193	0.056	<0.001^#^	0.600	0.752	0.003
**Isoleucine**	52.5	±	13.4	63.8	±	15.0	11.3	±	24.2	30.0	±	0.0∗	63.8	±	15.0	33.8	±	15.0^†^	<0.001^#^	0.375	<0.001^#^	0.571	0.004^#^	0.250
**Valine**	67.5	±	20.5	43.1	±	15.4	−24.4	±	19.7	63.8	±	15.0	50.6	±	14.4	−13.1	±	18.9	0.695	0.005	<0.001^#^	0.503	0.109	0.083
**Histidine**	48.8	±	15.0	46.9	±	15.4	−1.9	±	20.4	54.4	±	12.1	52.5	±	13.4	−1.9	±	20.4	0.109	0.083	0.607	0.009	1.000	<0.001
**Lysine**	60.0	±	0.0	54.4	±	12.1	−5.6	±	12.1	60.0	±	0.0	39.4	±	14.4∗	−20.6	±	14.4^†^	0.003^#^	0.254	<0.001^#^	0.510	0.003^#^	0.254
**Methionine**	41.3	±	15.0	35.6	±	12.1	−5.6	±	16.3	31.9	±	13.3	28.1	±	13.3	−3.8	±	24.2	0.010^#^	0.203	0.209	0.052	0.799	0.002
**Phenylalanine**	56.3	±	10.2	41.3	±	15.0	−15.0	±	19.0	60.0	±	0.0	46.9	±	24.4	−13.1	±	24.4	0.220	0.050	0.001^#^	0.306	0.810	0.002
**Threonine**	54.4	±	12.1	52.5	±	13.4	−1.9	±	20.4	54.4	±	12.1	45.0	±	15.5	−9.4	±	23.8	0.164	0.063	0.161	0.064	0.346	0.030
**Tryptophan**	41.3	±	15.0	37.5	±	23.2	−3.8	±	30.7	63.8	±	15.0∗	46.9	±	15.4	−16.9	±	18.9	0.001^#^	0.320	0.029^#^	0.148	0.156	0.066
**BCAA**	60.0	±	7.3	48.8	±	10.9	−11.3	±	12.6	52.5	±	10.0∗	53.1	±	10.8	0.6	±	13.4^†^	0.555	0.012	0.028^#^	0.151	0.015^#^	0.182
**EAA**	53.5	±	4.1	46.0	±	6.2	−7.5	±	6.1	53.5	±	4.8	46.5	±	7.1	−7.1	±	7.3	0.897	0.001	<0.001^#^	0.554	0.860	0.001
**Total amino acid**	53.1	±	3.7	51.0	±	3.9	−2.1	±	5.4	53.4	±	3.4	49.2	±	6.3	−4.2	±	6.8	0.525	0.014	0.007^#^	0.222	0.338	0.031

Data are shown as mean ± SD. Treatment effect was analyzed by unpaired Student's *t*-test. ∗*p* < 0.05. ^#^ presented a significant effect by repeated measures ANOVA (*p* < 0.05). The changes on Tmax were analyzed using the Mann-Whitney *U* test. ^†^*p* < 0.05.

### 3.6 Effect of administrating pea protein in combination with TWK10 on gut microbiota composition

**Fig. 4:**
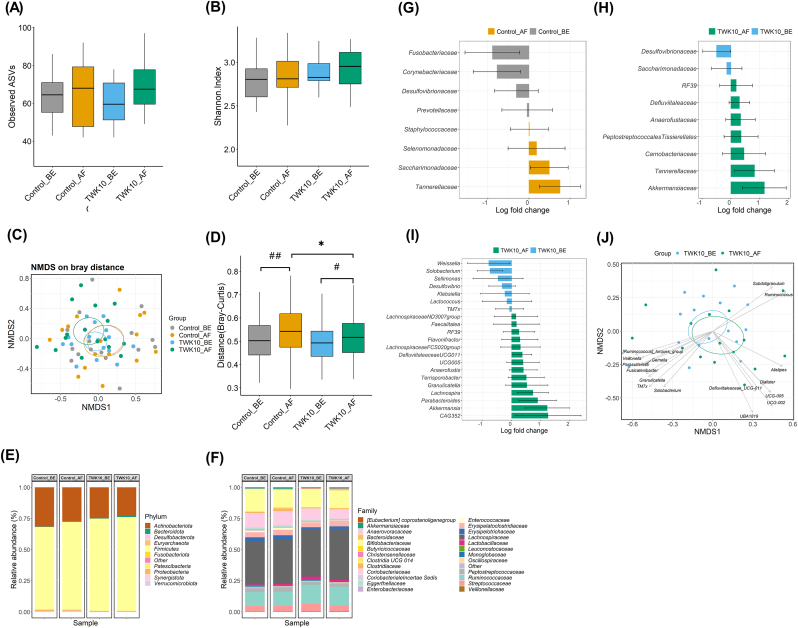
**Alpha and beta diversity of microbial communities.** (A, B) Box plots illustrate the differences in alpha-diversity indices (observed ASVs and Shannon index) between the control and TWK10 groups before and after administration, (C) NMDS ordinations based on Bray-Curtis distances between all samples at the ASV level. Differences on the NMDS plot between the values of each group were analyzed by PERMANOVA, and (D) Box plots show within treatment group variation of beta diversity measures for Bray-Curtis dissimilarity. Comparison of bacterial abundance between before and after administration in the control and TWK10 groups. The bacterial composition is based on the (E) top 10 phyla and (F) top 25 families of bacteria in all samples. Others represent the remaining phyla or families with lower relative abundance. Differential abundance analyses at the family level between before and after administration in the control (G) and TWK10 (H) groups by ANCOM-BC. Dynamic signature of gut microbiota components in the TWK10 group. (I) Differential abundance analyses at the genus level between before and after administration in the TWK10 group by ANCOM-BC. (J) Arrows show genera significantly correlated with the NMDS axes, which were determined by the envfit function of the R package vegan, with a cut-off for plotted results of *p* < 0.01 and R2 > 0.3. The direction of the arrow represents the correlation with changes in community composition, and the length of the arrow represents R^2^ values. Differences between before and after administration for each group were analyzed by the Wilcoxon signed-rank test. ^#^*p* < 0.05, ^##^*p* < 0.01. Treatment effect was analyzed by Mann-Whitney *U* test. ∗*p* < 0.05. ASV: Amplicon Sequence Variants.

To assess the impact of TWK10 administration on gut microbial composition, we analyzed the composition, abundance, and function of gut microbiota in fecal samples from the subjects using high-throughput sequencing of the V3-V4 region of 16S rRNA genes. The alpha-diversities were evaluated using the observed ASVs (Fig. 4A) and Shannon index (Fig. 4B). The observed ASVs ranged from 30 to 97 in all groups, and no significant differences were observed between before and after administration in the control and TWK10 groups (Fig. 4A). The Shannon index ranged from 1.87 to 3.34, and no significant differences were observed between before and after administration in the control and TWK10 groups (Fig. 4B). Additionally, NMDS based on Bray-Curtis distances (Fig. 4C) revealed no significant qualitative differences in gut microbiota compositions between before and after administration in the control (PERMANOVA, *p* = 0.969) and TWK10 groups (PERMANOVA, *p* = 0.931). We further assessed the change in microbiome composition by calculating the Bray-Curtis distances (Fig. 4D). The results showed significantly higher within-group variability in the control (*p* < 0.001) and TWK10 groups (*p* = 0.026) after administration compared with before administration. However, the TWK10 group had a significantly lower Bray-Curtis distance after administration compared to the control group after administration (*p* = 0.015).

The fecal microbiota of both control and TWK10 groups (before and after administration) consisted of five predominant phyla, including *Actinobacteriota*, *Bacteroidota*, *Firmicutes*, *Proteobacteria*, and *Verrucomicrobia* (Fig. 4E). The ANCOMBC algorithm was performed to accurately identify the impact of TWK10 on gut microbes at different taxon levels. The analysis revealed that the absolute abundance of *Desulfobacterota* (adjusted *p* < 0.0001) and *Fusobacteriota* (adjusted *p* < 0.0001) was significantly decreased in the control group after administration compared to before administration (Fig. S2A and Table S6). However, in the TWK10 group after administration, the absolute abundance of *Patescibacteria* (adjusted *p* < 0.0001) and *Verrucomicrobia* (adjusted *p* < 0.0001) was significantly increased, while *Desulfobacterota* (adjusted *p* < 0.0001) was significantly decreased compared to before administration (Fig. S2B and Table S7). At the family level, the top 10 predominant bacteria in subjects were *Lachnospiraceae*, *Ruminococcaceae*, *Bifidobacteriaceae*, *Coriobacteriaceae*, *Streptococcaceae*, *Erysipelatoclostridiaceae*, *Peptostreptococcaceae*, *Lactobacillaceae, Erysipelotrichaceae* and *Eubacterium coprostanoligenes* group (Fig. 4F and S2C). When compared with before administration, subjects with TWK10 administration displayed an increased abundance of *Akkermansiaceae*, *Tannerellaceae*, *Carnobacteriaceae*, *Peptostreptococcales-Tissierellales*, *Anaerofustaceae*, *Defluviitaleaceae* and *RF39* (Fig. 4H and Table S7). However, in comparison to the control group before administration, the family *Tannerellaceae*, *Saccharimonadaceae*, *Selenomonadaceae,* and *Staphylococcaceae* were significantly more abundant in the control group after administration (Fig. 4G and Table S6). At the genus level, TWK10 administration significantly increased the absolute abundance of 14 genera from *Firmicutes* and *Verrucomicrobia* in subjects (Fig. 4I and Table S7). The absolute abundance of genera *Defluviitaleaceae UCG-011* and *UCG-005* significantly increased and were positively correlated to the cluster of the TWK10 group after administration, while *Solobacterium* and *TM7x* significantly increased and were positively correlated to those in the TWK10 group before administration (Fig. 4I and **J**).

### 3.7 Correlation between the gut microbiota and TWK10-Mediated functional performance

**Fig. 5:**
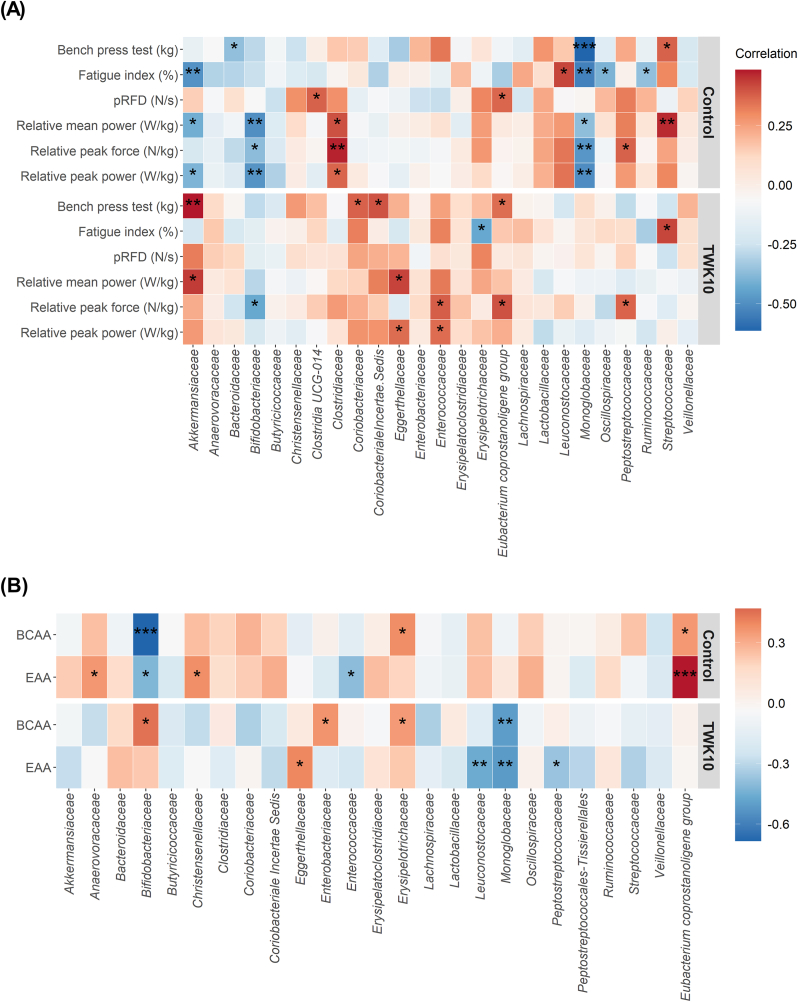
Heatmap showing Spearman correlations between Bacterial Taxa and Physiological Measures of Subjects in the Control and TWK10 Groups. (A) Correlations were examined between bacterial taxa (at family levels) and exercise performance indices, and (B) total BCAA and total EAA. The degree of correlation is represented by color hue: orange represents positive correlation; blue represents negative correlation. Significant values are denoted as ∗*p* < 0.05, *p* < 0.01 and ∗∗∗*p* < 0.001. (For interpretation of the references to color in this figure legend, the reader is referred to the Web version of this article.)

We examined the relationships between specific gut bacteria (top 25) and performance measures using Spearman's correlation coefficients (Fig. 5). In the TWK10 group, we found significant positive correlations between the relative abundance of *Akkermansiaceae* and the bench press test and relative mean power. *Eggethellaceae* showed positive correlations with relative peak power and relative mean power. Relative peak force was negatively correlated with *Bididobacteriaceae* and positively correlated with *Enterococcaceae*, *Eubacterium coprostanoligenes* group, and *Peptostreptococcaceae*. Additionally, the fatigue index was negatively correlated with *Erysipelotrichaceae* and positively correlated with *Streptococceae* (Fig. 5A). We also examined the potential connections between gut bacteria and amino acid absorption rates. BCAA showed positive correlations with *Bididobacteriaceae*, *Enterobacteriaceae*, and *Erysipelotrichaceae*, but a negative correlation with *Monglobaceae*. Furthermore, EAA displayed an inverse correlation with *Leuconostocaceae*, *Monglobaceae*, and *Peptostreptococcaceae*, while showing a positive correlation with *Eggerthellaceae*.

The network map (Fig. S3) supported the observed associations, showing positive correlations among TWK10-enriched bacteria (Fig. 5B). For example, *Anaerofustaceae* showed positive correlations with *Oscillospiraceae*, *Erysipelotrichaceae*, and *Christensenellaceae*. *Carnobacteriaceae* was positively correlated with *Lachnospiraceae* and *Eggerthellaceae*. *Tannerellaceae* showed a positive correlation with *Bacteroidaceae*, which displayed a significant increasing trend (*p* = 0.073) in abundance in the TWK10 group after administration (Fig. S2C). We also found that *Bacteroidaceae* had positive associations with several bacteria belonging to phylum *Firmicutes*, including *Oscillospiraceae* and *Erysipelotrichaceae* (Fig. S3D). Meanwhile, TWK10-diminished *Saccharimonadaceae* negatively co-occurred with *Erysipelotrichaceae* and *Anaerofustaceae* (Fig. S3D).

### 3.8 Potential functional annotations of gut microbiota in the TWK10 group after administration

**Fig. 6:**
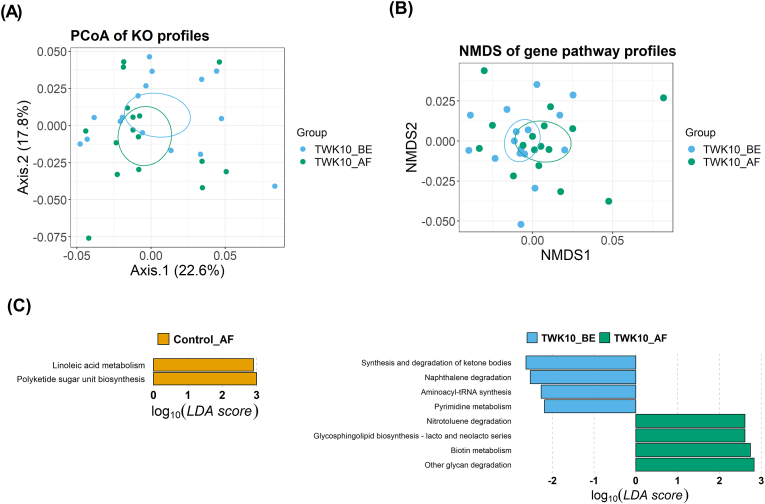
Differences in Functional Orthologs and KEGG Pathways of the Fecal Microbial Community in the TWK10 Group. (A) Principal coordinate analysis (PCoA) of the KO profiles, (B) KEGG pathways that discriminated the fecal microbiomes and (C) Corresponding bar plot of KEGG pathway function differential analysis comparing samples from control after administration (Control_AF, *n* = 16) versus control before administration (Control_BE, *n* = 16) and TWK10 after administration (TWK10_AF, *n* = 16) versus TWK10 before administration (TWK10_BE, *n* = 16).

We performed PICRUSt2 analyses to investigate the variation in the metabolic potential of the gut microbiota within the TWK10 group. A total of 157 KEGG pathways were predicted across both the control and TWK10 groups. To assess the functional similarities of the gut microbiota among individuals, we conducted PCoA based on the relative abundance of KOs and KEGG pathways. The results indicated no significant differences in the KO profiles (*R2* = 0.034, *p* = 0.361) (Fig. 6A) and pathway profiles (Fig. 6B) (*R2* = 0.034, *p* = 0.372) between before and after administration in the TWK10 group. Through LEfSe analysis, we identified four enriched pathways (KEGG level 3) in the TWK10 group after administration (Fig. 6C). These pathways were Nitrotoluene degradation, Glycosphingolipid biosynthesis - lacto and neolacto series, Biotin metabolism, and other glycan degradation. In contrast, in the control group, the administration of pea protein induced changes in Linoleic acid metabolism and Polyketide sugar unit biosynthesis.

## 4 Discussion

Maintaining a positive protein balance in conjunction with resistance exercise has been shown to promote muscle protein synthesis and repair. Additionally, probiotics have been previously suggested to improve muscle mass and delay fatigue (Huang et al., 2019). In this study, after 4 weeks of resistance training with pea protein supplementation, the TWK10 probiotic group showed significant increases in blood concentrations and absorption of essential amino acids (EAAs) and branched-chain amino acids (BCAA), resulting in enhanced muscle strength and power. Although there were no significant changes in overall body composition, muscle and fascia thickness were significantly increased in the TWK10 group. Moreover, analysis of the gut microbiota revealed that TWK10 supplementation increased the richness of intestinal microorganisms, which positively correlated with amino acid absorption rates and exercise performance.

Protein intake is a critical factor in regulating muscle mass and sustaining anabolic stimulation by increasing the circulation of EAAs (Wolfe, 2002). In the past, it was believed that certain compounds like tannins and phytic acids in plant-based proteins could negatively impact protein digestibility, reducing anabolic properties and postprandial amino acid absorption rates (Berrazaga et al., 2019). A previous meta-analysis comparing long-term studies (≥6 weeks) of soy protein or whey protein supplementation combined with resistance training found no significant differences in gains in muscle mass and strength between groups (Messina et al., 2018). In our study, both groups showed a trend towards increased muscle mass and decreased body fat percentage after 4 weeks of intervention, but these changes did not reach statistical significance (Table S5). Both groups received pea protein and resistance training, potentially contributing to the observed increase in muscle mass. However, the key difference between the two groups in this study was the addition of TWK10, which in previous studies has shown significant increases in muscle mass in both mice and humans without additional exercise training. Notably, in human studies, a high dose of 9 × 10^10^ CFU/day has achieved significant effects, whereas our study used a dose of 1 × 10^10^ CFU/day (Chen et al., 2016; Huang et al., 2019). Therefore, whether exercise training can enhance the benefits of TWK10 supplementation or if a higher TWK10 dosage is needed to significantly increase muscle mass requires further investigation, as a larger CFU count does not always equate to higher efficacy. Additionally, it's worth considering that changes in muscle structure typically occur between the fourth and eighth week of resistance training, suggesting that extending the intervention time might reveal more significant differences (Gondin et al., 2005).

Previous studies using meta-analysis and meta-regression methods have shown that protein supplementation significantly increases strength, lean body mass, and muscle cross-sectional area (Morton et al., 2018). In a past study that compared 12 consecutive weeks of resistance training with supplementation of placebo, whey protein, and pea protein, the pea protein supplementation group showed a significant increase in muscle thickness, even higher than the whey protein group, though this difference did not reach statistical significance (Babault et al., 2015). Our study also showed a trend towards increased muscle mass, which was not significant, but there was a significant increase in muscle thickness and fascia in the left and right biceps and right quadriceps after 4 weeks of TWK10 and pea protein supplementation combined with resistance training (Fig. 2). Increased muscle thickness may also be accompanied by increased fascial thickness, as higher muscle strength and body weight require thicker connective tissue to withstand and transmit greater forces (Wilke et al., 2019). Muscle strength is generally determined by the product of muscle quality and muscle strength relative to muscle volume, making increasing muscle thickness fundamental for achieving strength gains (Ikai and Fukunaga, 1970). A previous animal experiment showed that supplementing pea peptide for 8 consecutive weeks combined with resistance training significantly enhanced the expression of insulin-like growth factor 1 receptor and AMP-activated protein kinase, while significantly reducing the expression of myostatin in skeletal muscle, thereby increasing the rat's muscle cross-section and muscle strength (Jia et al., 2022). Another two-week study of *Bacillus coagulans* supplemented with 20 g of casein showed a significant improvement in anaerobic performance as assessed by the Wingate test, with a trend toward increased vertical jump strength (Jäger et al., 2016). These findings appear to support the significant increases in 1-RM bench press strength, isometric muscle strength, and anaerobic power output observed in our study, which can be attributed to increased muscle thickness. Furthermore, in our results, the group containing TWK10 showed a more significant increase in strength and a significantly higher difference value compared to the group without TWK10 (Fig. 3).

EAAs are essential to significantly stimulate muscle protein synthesis (MPS) (Volpi et al., 2003). Muscle proteins provide a continuous supply of necessary precursors from EAAs to other tissues, maintaining protein synthesis rates and activating protein synthesis mechanisms in skeletal muscle tissue. However, the rate of MPS is generally lower than the rate of muscle protein breakdown. Thus, in the absence of exogenous amino acid intake, a state of synthesis metabolism does not occur (Wolfe, 2017). Amino acids must be absorbed to be utilized by the body, making amino acid absorbability a critical factor in assessing protein quality (Trommelen et al., 2021). The postprandial increase in plasma amino acid concentration, after dietary proteins are digested, and amino acids are absorbed and released into circulation, is considered a representative indicator of protein digestion and amino acid absorption (Gorissen et al., 2016). While most dietary amino acids and peptides from the upper gastrointestinal tract are readily absorbed, large amounts of proteins, peptides, some amino acids, and other nitrogenous metabolites enter the large intestine and are further digested by bacterial enzymes and surviving trypsins and peptidases (van der Wielen et al., 2017).

Previous studies have noted that the gut diversity of athletes is high and positively associated with many amino acid and carbohydrate metabolic pathways, including those involved in metabolizing BCAAs (Clarke et al., 2014; Petersen et al., 2017). This suggests that the gut microbiota can promote host protein anabolism by increasing amino acid bioavailability, insulin secretion, and skeletal muscle response (Ticinesi et al., 2019). Certain probiotic strains possess proteolytic properties that increase digestive enzyme production and host protein utilization, such as *Bacillus coagulans, Lactiplantibacillus plantarum, Lacticaseibacillus rhamnosus, Streptococcus thermophilus, Lacticaseibacillus paracasei*, etc. (Jäger et al., 2018; Kieliszek et al., 2021). Previous animal experiments have shown that probiotic supplementation increases L. plantarum and bifidobacteria in the gut, alters the microbiome, restores gut homeostasis, and influences muscle size through the muscle-gut axis (Bindels et al., 2016). In this study, supplementation with pea protein combined with resistance training for 4 weeks significantly increased the AUC and maximum concentration of BCAAs, EAAs, and total amino acids compared to before the intervention, and these improvements were even more significant compared to the control group (Table 1, Table 2). Additionally, leucine concentration in the TWK10 group was significantly increased, which may explain the observed increases in muscle thickness and strength. Leucine is known to be sensed by sestrin2, promoting the translocation of the mammalian target of rapamycin complex 1 to the lysosomal membrane, and activating downstream signaling pathways involved in MPS control. It possesses the strongest anabolic properties and is a key factor in promoting muscle hypertrophy (Saxton et al., 2016).

Gut microbes were further analyzed to understand the mechanism of action of TWK10 on amino acid absorption. Although no advantage in alpha diversity was observed after TWK10 administration in this study, a significant increase in specific bacterial phyla was still observed in the TWK10 group (Fig. 4A–D). Among them, the absolute abundance of *Verrucomicrobia*, including one of the *Akkermansiaceae* family, was significantly increased after TWK10 administration (Fig. 4E–H). *Akkermansiaceae* is known to improve intestinal health by adhering to intestinal epithelial cells and enhancing the integrity of the intestinal epithelial monolayer, and it is related to the production of short-chain fatty acids (SCFAs) (Barton et al., 2018). Previous animal experiments have shown that *Akkermansia muciniphila* significantly improved muscle performance in mice after 4 weeks of administration (Byeon et al., 2022), which is consistent with our findings of a significant positive correlation between the relative abundance of *Akkermansiaceae* and relative mean strength in the bench press test after TWK10 administration (Fig. 5).

TWK10 administration significantly increased the abundance of some bacterial families known to play an important role in amino acid production and de novo biosynthesis, as well as in the utilization of amino acids for reactive fermentation to produce SCFAs (Neis et al., 2015). While SCFAs are generally considered markers of glycolytic fermentation, our study also highlights that propionate and butyrate are produced during amino acid fermentation (Van den Abbeele et al., 2022). Additionally, we found that TWK10 administration increased the relative abundance of the *Desulfovibrio* family (Fig. 4E–H), which is thought to contain genes responsible for amino acid synthesis. Furthermore, we observed positive correlations between BCAA and *Bifidobacteriaceae*, *Enterobacteriaceae*, and *Erysipelotrichaceae* (Fig. 5). Previous animal studies have shown that increased abundance of Akkermansia and Bifidobacteria in the gut and decreased Enterobacteriaceae favored increased BCAA levels (Yang et al., 2016). Another study found that Erysipelotrichaceae was negatively correlated with phenylalanine and leucine (Li et al., 2017). However, *Enterobacteriaceae*'s role may differ between animals and humans, and *Danobacteriaceae* participates in butyric acid synthesis by metabolizing specific amino acids (Estaki et al., 2016), which may explain the negative correlation between *Erysipelotrichaceae* and the fatigue index observed in our study (Fig. 5). Overall, pea protein combined with resistance training and supplemented with TWK10 for 4 weeks increased the abundance of specific gut bacteria, leading to increased amino acid absorption and improved muscle performance. However, many of the bacterial families that increased in abundance are still unknown for their role in amino acid uptake or muscle strength performance, and further research is needed to identify the relevant bacterial species and mechanisms involved.

This study had several limitations including: (1) This study mainly compares the effect of TWK10 on improving amino acid absorption rate and exercise performance, so male and female subjects are included in the same group for comparison. While known strength or physiological differences between male and female subjects may result in large standard deviations, the benefit of treatment between different samples can be observed in different populations. In the future, more subjects can be included to further compare the effect and mechanism of this trial sample in male and female subjects. (2) The participants in this study were all non-athletic major students from the same university. While their living conditions are similar, which helps reduce effects in the study, it may not be representative of different ethnic groups. (3) This is the first study to combine supplementation with *L. plantarum* and plant protein, so we evaluated the effectiveness of a single dose and a four-week intervention. However, whether higher doses of probiotics, protein intake, or even longer intervention are needed to achieve better results requires further comparative verification. (4) At present, there are still many bacteria in the intestinal tract that have not been identified, and the regulation of intestinal microbes on amino acid absorption still needs more verification. (5) Although the main purpose of this study is to explore the relationship between amino acid absorption rate and muscle mass and strength, it may be the most direct method to discover the underlying cellular mechanism through muscle biopsy. However, collecting muscle samples in our country is a medical act, which needs to be performed by professional physicians and more stringent scrutiny. In addition, it is likely that this will also cause a greater degree of harm to the subject. Therefore, it needs to be verified by other methods in the future. (6) Compared with deep sequencing, which can produce a large amount of sequence data and reveal the composition and function of microbial communities, shallow sequencing is less expensive, fast and provides sufficient information. Although 16S rRNA sequencing can quickly and accurately describe the composition of microbial communities, it cannot provide information about function and the entire genome, and it has limitations in the classification and resolution of microbial species. In future studies, we will explore more subtle microbial community changes when conducting further research and experimental design, thereby improving our understanding and description of microbial communities.

## 5 Conclusion

In conclusion, our study demonstrates that supplementation of pea protein in combination with resistance training and *L. plantarum* TWK10 can significantly increase the area under the curve (AUC) and maximum concentration of BCAAs, EAAs, and total amino acids after four weeks of administration. Additionally, there is a trend towards increased muscle mass, and a significant improvement in muscle thickness, resulting in enhanced maximum muscle strength, explosive power, and anaerobic power. In terms of practical applications, this result will help vegetarians and even athletes improve the effectiveness of protein intake to enhance amino acid absorption and muscle synthesis. Not only does it have the benefits of probiotics or protein, but when the two are combined, it creates greater benefits as a new strategy for sports nutrition supplements. Future research should focus on exploring the specific bacterial species that increase after TWK10 administration, understanding the underlying mechanisms, and conducting further investigations to compare the effects in different genders and with varying doses.

## CRediT authorship contribution statement

**Mon-Chien Lee:** Writing – original draft, Writing – review & editing, Investigation, Formal analysis, Validation, Data curation. **Chun-Hui Chiu:** Methodology, Validation. **Yi-Chu Liao:** Formal analysis, Methodology. **Yi-Chen Cheng:** Conceptualization, Formal analysis. **Chia-Chia Lee:** Conceptualization, Project administration. **Chin-Shan Ho:** Formal analysis, Methodology. **Yi-Ju Hsu:** Investigation, Methodology. **Hsiao-Yun Chang:** Formal analysis, Investigation. **Jin-Seng Lin:** Conceptualization, Supervision. **Chi-Chang Huang:** Conceptualization, Data curation, Validation, Writing – review & editing.

## Ethical approval and consent to participate

The study protocol was approved by the Institutional Review Board of Landseed International Hospital (Taoyuan, Taiwan; LSHIRB number 21-035-A2) according to the ethical standards laid down in the 1964 Declaration of Helsinki and its later amendments. All participants gave their informed consent prior to their inclusion in the study.

## Publication statement

This manuscript has not been published elsewhere and has not been submitted simultaneously for publication elsewhere.

## Funding

This study was funded by the 10.13039/100019767University–Industry Cooperation Fund, National Taiwan Sport University, Taoyuan, Taiwan (NTSU No.1101064).

## Declaration of competing interest

The authors declare the following financial interests/personal relationships which may be considered as potential competing interests: Yi-Chu Liao reports a relationship with Culture Collection & Research Institute, SYNBIO TECH INC that includes: employment. Yi-Chen Cheng reports a relationship with Culture Collection & Research Institute, SYNBIO TECH INC that includes: employment. Chia-Chia Lee reports a relationship with Culture Collection & Research Institute, SYNBIO TECH INC that includes: employment. Jin-Seng Lin reports a relationship with Culture Collection & Research Institute, SYNBIO TECH INC that includes: employment. If there are other authors, they declare that they have no known competing financial interests or personal relationships that could have appeared to influence the work reported in this paper.

## Data Availability

Data will be made available on request.
