# Supplementary material for: Gut microbiota modulation and amino acid absorption by Lactiplantibacillus plantarum TWK10 in pea protein ingestion: TWK10 boosts hut microbiota, amino acid uptake
Source: Curr Res Food Sci. 2024 Nov 9;9:100917. doi: 10.1016/j.crfs.2024.100917 (PMC11613169; doi:10.1016/j.crfs.2024.100917)
Supplement: Multimedia component 1 [file mmc1.docx]

**Table S1.** General characteristics of the subjects

| **Basic information** | **Control**  ***n* = 16 (8 male, 8 female)** | **TWK10**  ***n* = 16 (8 male, 8 female)** |
| --- | --- | --- |
| Age (years old) | 25±6 | 23±3 |
| Height (cm) | 166±9 | 168±10 |
| Weight (kg) | 66±8 | 63±11 |
| SBP (mmHg) | 115±6 | 117±6 |
| DBP (mmHg) | 65±6 | 69±6 |
| HR (Bpm) | 71±7 | 68±9 |
| **Exercise habits** |  |  |
| **How many days do you exercise per week?** |  |  |
| 1 day/week | 0 | 0 |
| 2-3 days/week | 9 | 9 |
| 4-7 days/week | 7 | 7 |
| **How many hours do you exercise per day?** |  |  |
| <1 hour/day | 0 | 0 |
| 1-2 hours/day | 11 | 10 |
| >2 hours/day | 5 | 6 |
| **How long does your aerobic exercise last/day?** |  |  |
| <30 minute/day | 4 | 6 |
| >30 minute/day | 12 | 10 |
| **How many hours does your weight-training last/day?** |  |  |
| <1 hour/day | 7 | 6 |
| 1-2 hours/day | 9 | 10 |
| >2 hours/day | 0 | 0 |

Data are presented as mean ± SD on basic information. In exercise habits, the numbers represented the number of people.

**Table S2.** MRM conditions for 23 amino acids and isotope internal standard

| **Analyte** | **M.W.**  **(g/mol)** | **Quantitative ion** | | |
| --- | --- | --- | --- | --- |
|  |  | **Precursor ion (*m/z*) >**  **Product ion (*m/z*)** | **Cone**  **voltage (V)** | **Collision energy (eV)** |
| Alanine | 89.1 | 260.2 > 171.1 | 30 | 60 |
| Alpha Aminobutyric Acid | 103.1 | 274.2 > 171.10 | 30 | 60 |
| Arginine | 174.2 | 345.1 > 171.10 | 30 | 20 |
| Asparagine | 132.1 | 303.1 > 171.1 | 30 | 60 |
| Aspartic acid | 133.1 | 304.2> 171.1 | 30 | 60 |
| Citrulline | 175.2 | 346.2 > 171.1 | 30 | 20 |
| Cysteine | 240.3 | 291.1 > 171.1 | 30 | 15 |
| L-Ornithine | 132.2 | 237.10 > 17.1 | 30 | 15 |
| Glutamine | 146.1 | 317.1 > 171.1 | 30 | 60 |
| Glycine | 75.1 | 246.1 > 171.1 | 30 | 60 |
| Proline | 115.2 | 286.1 > 117.1 | 30 | 60 |
| Serine | 105.1 | 276.1 > 171.1 | 30 | 60 |
| Taurine | 125.2 | 296.2 > 171.1 | 30 | 20 |
| Tyrosine | 181.2 | 352.1 > 171.1 | 30 | 60 |
| Leucine | 131.2 | 302.1 > 171.1 | 30 | 60 |
| Isoleucine | 131.2 | 302.1 > 171.1 | 30 | 60 |
| Valine | 117.2 | 288.2 > 171.1 | 30 | 60 |
| Histidine | 155.1 | 326.2 > 171.1 | 30 | 20 |
| Lysine | 146.2 | 244.1 > 171.1 | 30 | 15 |
| Methionine | 149.2 | 320.2 > 171.1 | 30 | 60 |
| Phenylalanine | 165.2 | 336.1 > 171.1 | 30 | 60 |
| Threonine | 119.1 | 290.2 > 171.1 | 30 | 60 |
| Tryptophan | 204.3 | 375.2 > 171.1 | 30 | 60 |
| Alanine ^13^C_3_, ^15^N | 93.1 | 264.2 >171.1 | 30 | 20 |
| Arginine ^13^C_6_, ^15^N_4_ | 184.2 | 355.2 >171.1 | 30 | 20 |
| Asparagine ^13^C_4_, ^15^N_2_ | 138.1 | 309.2 >171.1 | 30 | 20 |
| Aspartic Acid ^13^C_4_, ^15^N | 138.1 | 309.2 >171.1 | 30 | 20 |
| Cystine ^13^C_6_, ^15^N_2_ | 248.2 | 295.1 >171.1 | 30 | 15 |
| Glutamic Acid ^13^C_5_, ^15^N | 153.1 | 324.2 >171.1 | 30 | 20 |
| Glutamine ^13^C_5_, ^15^N_2_ | 153.1 | 324.2 >171.1 | 30 | 20 |
| Glycine ^13^C_2_, ^15^N | 78.1 | 249.2 >171.1 | 30 | 20 |
| Histidine ^13^C_6_, ^15^N_3_ | 164.1 | 335.2 >171.1 | 30 | 20 |
| Isoleucine ^13^C_6_, ^15^N | 138.1 | 309.2 >171.1 | 30 | 20 |
| Leucine ^13^C_6_, ^15^N | 138.1 | 309.2 >171.1 | 30 | 20 |
| Lysine ^13^C_6_,^15^N_2_ | 154.1 | 248.1 >171.1 | 30 | 15 |
| Methionine ^13^C_5_, ^15^N | 155.1 | 326.2 >171.1 | 30 | 20 |
| Phenylalanine ^13^C_9_, ^15^N | 175.1 | 346.2 >171.1 | 30 | 20 |
| Proline ^13^C_5_, 15N | 121.1 | 292.2 >171.1 | 30 | 20 |
| Serine ^13^C_3_, 15N | 109.1 | 280.2 >171.1 | 30 | 20 |
| Threonine ^13^C_4_, 15N | 124.1 | 295.2 >171.1 | 30 | 20 |
| Tryptophan ^13^C_11_, 15N_2_ | 217.1 | 388.2 >171.1 | 30 | 20 |
| Tyrosine ^13^C_9,_ 15N | 191.1 | 362.2 >171.1 | 30 | 20 |
| Valine ^13^C_5_, 15N | 123.1 | 294.2 >171.1 | 30 | 20 |

**Table S3.** Retention time, calibration curves and linearity assay of 23 amino acids

| **Analyte** | **Retention time (mins)** | **slope** | **intercept** | **Calibration range (µM)** | ***r*** |
| --- | --- | --- | --- | --- | --- |
| Alanine | 2.61 | 0.018 | -0.017 | 5-4000 | 0.9969 |
| Alpha Aminobutyric Acid | 2.92 | 0.068 | -0.040 | 5-1000 | 0.9982 |
| Arginine | 2.18 | 0.987 | -0.215 | 5-1000 | 0.9999 |
| Asparagine | 2.17 | 0.056 | 0.060 | 5-1000 | 0.9984 |
| Aspartic Acid | 2.38 | 0.042 | 0.091 | 5-1000 | 0.9979 |
| Citrulline | 2.40 | 0.135 | -0.053 | 5-1000 | 0.9994 |
| Cystine | 2.87 | 2.608 | -3.756 | 2.5-500 | 0.9957 |
| L-Ornithine | 2.72 | 0.319 | 0.123 | 5-4000 | 0.9998 |
| Glutamine | 2.27 | 0.109 | -0.489 | 5-4000 | 0.9950 |
| Glycine | 2.34 | 0.012 | -0.036 | 5-4000 | 0.9972 |
| Proline | 2.74 | 0.038 | 0.105 | 5-1000 | 0.9984 |
| Serine | 2.27 | 0.036 | -0.054 | 5-4000 | 0.9995 |
| Taurine | 2.26 | 0.355 | -0.028 | 5-1000 | 0.9999 |
| Tyrosine | 3.24 | 0.192 | 0.141 | 5-4000 | 0.9994 |
| Leucine | 5.45 | 0.079 | -0.089 | 5-4000 | 0.9979 |
| Isoleucine | 5.20 | 0.085 | -0.210 | 5-4000 | 0.9994 |
| Valine | 3.61 | 0.055 | -0.038 | 5-4000 | 0.9999 |
| Histidine | 2.05 | 0.818 | -0.467 | 5-1000 | 0.9987 |
| Lysine | 2.88 | 0.967 | -0.138 | 5-1000 | 0.9994 |
| Methionine | 3.47 | 0.099 | 0.069 | 5-1000 | 0.9982 |
| Phenylalanine | 5.93 | 0.159 | -0.126 | 5-4000 | 0.9985 |
| Threonine | 2.51 | 0.048 | 0.009 | 5-4000 | 0.9999 |
| Tryptophan | 6.32 | 0.214 | -0.231 | 5-1000 | 0.9989 |

**Table S4**. Subject’s dietary intake before and after the 4 weeks pea protein combined with TWK10 administration.

| **Dietary intake** | **Control** | | | | | |  | **TWK10** | | | | | |
| --- | --- | --- | --- | --- | --- | --- | --- | --- | --- | --- | --- | --- | --- |
|  | **Before** | | | **After** | | |  | **Before** | | | **After** | | |
| Carbohydrate (g/day) | 179 | ± | 38 | 182 | ± | 34 |  | 179 | ± | 17 | 183 | ± | 22 |
| Protein (g/day) | 82 | ± | 7 | 100 | ± | 8^###^ |  | 84 | ± | 11 | 100 | ± | 12^###^ |
| Fat (g/day) | 56 | ± | 11 | 57 | ± | 11 |  | 53 | ± | 9 | 54 | ± | 10 |
| Total calorie (kcal/day) | 1542 | ± | 223 | 1640 | ± | 184^##^ |  | 1529 | ± | 139 | 1620 | ± | 165^###^ |

Data are presented as mean ± SD. Treatment effect was analyzed by unpaired Student’s *t*‐test. Differences between before and after administration for each group were analyzed by paired Student’s *t*‐test. ^##^ *p* < 0.01, ^###^ *p* < 0.001.

**Table S5.** Subject’s body composition before and after the 4 weeks pea protein combined with TWK10 administration.

| **Body**  **composition** | **Control** | | | | | | | | |  | **TWK10** | | | | | | | | |
| --- | --- | --- | --- | --- | --- | --- | --- | --- | --- | --- | --- | --- | --- | --- | --- | --- | --- | --- | --- |
|  | **Before** | | | **After** | | | **Change** | | |  | **Before** | | | **After** | | | **Change** | | |
| Body weight (kg) | 65.5 | ± | 8.4 | 65.1 | ± | 7.8 | -0.4 | ± | 1.6 |  | 62.8 | ± | 10.5 | 62.5 | ± | 10.3 | -0.2 | ± | 2.2 |
| BMI (kg/m^2^) | 23.7 | ± | 3.0 | 23.5 | ± | 2.6 | -0.2 | ± | 0.6 |  | 22.1 | ± | 2.7 | 21.9 | ± | 2.2 | -0.1 | ± | 0.8 |
| LBM (kg) | 45.9 | ± | 8.9 | 46.8 | ± | 9.3^##^ | 0.9 | ± | 1.0 |  | 43.6 | ± | 10.6 | 44.6 | ± | 9.8 | 1.1 | ± | 2.1 |
| FBM (%) | 26.6 | ± | 9.2 | 26.0 | ± | 9.3 | -0.6 | ± | 1.6 |  | 25.3 | ± | 7.0 | 24.6 | ± | 6.6 | -0.7 | ± | 1.4 |

Data are shown as mean ± SD. Treatment effect on body weight and LBM was analyzed by unpaired Student’s *t*-test, and Mann-Whitney *U* test was used for statistical comparison on BMI and FBM. Differences on body weight and LBM between before and after administration for each group were analyzed by paired Student’s *t*‐test, and Wilcoxon signed-rank test was used for statistical comparison on BMI and FBM. ^##^ *p* < 0.01. The changes on body weight, BMI, muscle mass, and fat mass were calculated as the difference between after and before administration and statistical significance was analyzed using the Mann-Whitney *U* test. BMI, body mass index. LBM, lean body mass, LBM = body weight – body weight × FBM. FBM, fat body mass.

**Table S6.** Summary of differential microbiota in the control group.

| **Taxa level** | **Taxa name** | **beta** | **W** | **q value** |
| --- | --- | --- | --- | --- |
| Phylum | *Desulfobacterota* | -0.122690609 | -0.219515441 | <0.0001 |
|  | *Fusobacteriota* | -0.706164446 | -1.072513045 | <0.0001 |
| Family | *Corynebacteriaceae* | -0.776495848 | -1.344580055 | <0.0001 |
|  | *Desulfovibrionaceae* | -0.306818576 | -0.576242193 | <0.0001 |
|  | *Fusobacteriaceae* | -0.890292412 | -1.335073548 | <0.0001 |
|  | *Prevotellaceae* | -0.044253811 | -0.072146442 | <0.0001 |
|  | *Saccharimonadaceae* | 0.493688836 | 1.068277186 | <0.0001 |
|  | *Selenomonadaceae* | 0.186117117 | 0.268100449 | <0.0001 |
|  | *Staphylococcaceae* | 0.009668896 | 0.021017768 | <0.0001 |
|  | *Tannerellaceae* | 0.756153301 | 1.531384157 | <0.0001 |
| Genus | *Acidaminococcus* | 0.547419984 | 1.206014005 | <0.0001 |
|  | *Anaerococcus* | -0.310030785 | -0.440761201 | <0.0001 |
|  | *Corynebacterium* | -0.825770382 | -1.407362877 | <0.0001 |
|  | *Eubacterium eligen group* | 1.045911861 | 1.582935790 | <0.0001 |
|  | *Fusobacterium* | -0.939566946 | -1.398331859 | <0.0001 |
|  | *Holdemania* | 0.452219897 | 0.810650562 | <0.0001 |
|  | *Klebsiella* | -0.679255594 | -0.871932590 | <0.0001 |
|  | *Megamonas* | 0.136842584 | 0.201214136 | <0.0001 |
|  | *Negativibacillus* | 0.376751676 | 0.841075134 | <0.0001 |
|  | *Parabacteroides* | 0.706878768 | 1.475478065 | <0.0001 |
|  | *Peptostreptococcus* | 0.569329554 | 1.069415385 | <0.0001 |
|  | *Prevotella* | -0.022440949 | -0.040220563 | <0.0001 |
|  | *Staphylococcus* | -0.039605637 | -0.086115971 | <0.0001 |
|  | *TM7x* | 0.444414303 | 0.943098360 | <0.0001 |

Beta in ANCOMBC results represented as log fold change. A positive or negative W-value indicates a decrease or increase in absolute abundance in the control group. p-values were adjusted using “Benjamini-Hochberg” correction and presented as q- values.

**Table S7.** Summary of differential microbiota in the TWK10 group.

| Taxa level | Taxa name | beta | W | q value |
| --- | --- | --- | --- | --- |
| Phylum | *Desulfobacterota* | -0.466421316 | -0.853112055 | <0.0001 |
|  | *Patescibacteria* | 0.061412102 | 0.100939626 | <0.0001 |
|  | *Verrucomicrobiota* | 1.186231273 | 1.436352967 | <0.0001 |
| Family | *Akkermansiaceae* | 1.157344464 | 1.525380854 |  |
|  | *Anaerofustaceae* | 0.333338610 | 0.652622018 | <0.0001 |
|  | *Carnobacteriaceae* | 0.452639245 | 0.611778601 | <0.0001 |
|  | *Defluviitaleaceae* | 0.293615027 | 0.817543076 | <0.0001 |
|  | *Desulfovibrionaceae* | -0.495308125 | -1.030292288 | <0.0001 |
|  | *Peptostreptococcales-Tissierellales* | 0.350481147 | 0.595207718 | <0.0001 |
|  | *RF39* | 0.180836133 | 0.319574568 | <0.0001 |
|  | *Saccharimonadaceae* | -0.144550541 | -0.273961971 | <0.0001 |
|  | *Tannerellaceae* | 0.814808462 | 1.184444451 | <0.0001 |
|  | *Akkermansiaceae* | 1.157344464 | 1.525380854 | <0.0001 |
| Genus | *Akkermansia* | 1.247524948 | 1.621285122 | <0.0001 |
|  | *Anaerofustis* | 0.423519095 | 0.891439587 | <0.0001 |
|  | *CAG352* | 1.273218106 | 1.113808054 | <0.0001 |
|  | *Defluviitaleaceae UCG011* | 0.383795512 | 1.149995439 | <0.0001 |
|  | *Desulfovibrio* | -0.514063222 | -1.207623353 | <0.0001 |
|  | *Faecalitalea* | 0.183643192 | 0.232749745 | <0.0001 |
|  | *Flavonifractor* | 0.157764749 | 0.246447641 | <0.0001 |
|  | *Granulicatella* | 0.542819729 | 0.742769911 | <0.0001 |
|  | *Klebsiella* | -0.214547428 | -0.255044142 | <0.0001 |
|  | *Lachnospira* | 0.750715400 | 1.388810311 | <0.0001 |
|  | *Lachnospiraceae FCS020 group* | 0.319269291 | 0.468363896 | <0.0001 |
|  | *Lachnospiraceae ND3007 group* | 0.163274054 | 0.217861912 | <0.0001 |
|  | *Lactococcus* | -0.14317582 | -0.166723725 | <0.0001 |
|  | *Parabacteroides* | 0.904988946 | 1.393068611 | <0.0001 |
|  | *RF39* | 0.271016617 | 0.495026198 | <0.0001 |
|  | *Sellimonas* | -0.452866161 | -0.522965580 | <0.0001 |
|  | *Solobacterium* | -0.729575936 | -1.662166346 | <0.0001 |
|  | *TM7x* | -0.054370056 | -0.111084847 | <0.0001 |
|  | *UCG005* | 0.381471697 | 0.691846584 | <0.0001 |
|  | *Weissella* | -0.775930767 | -1.058442378 | <0.0001 |

Beta in ANCOMBC results represented as log fold change. A positive or negative W-value indicates a decrease or increase in absolute abundance in the TWK10 group. p-values were adjusted using “Benjamini-Hochberg” correction and presented as q- values.


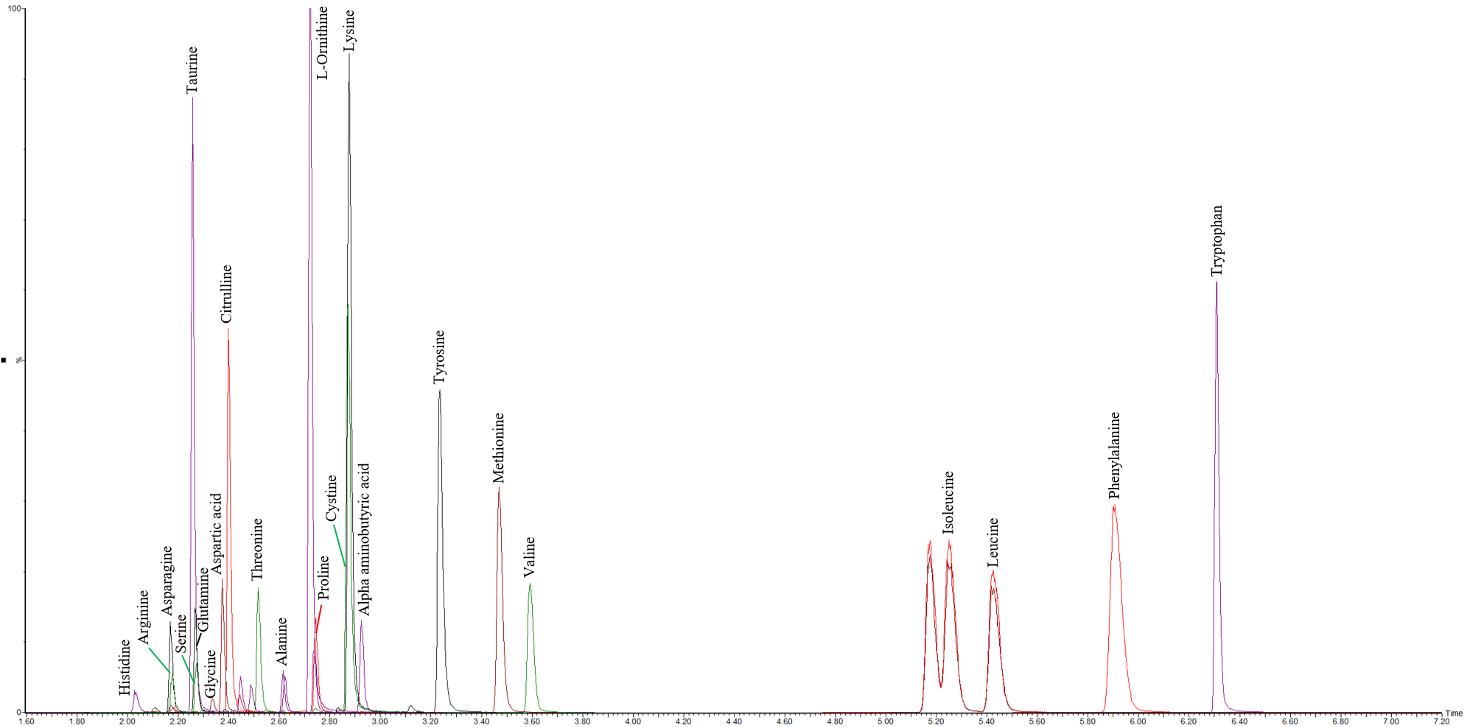


**Figure S1.** LC-MS/MS analysis of 23 amino acids.





**Fig S2.** Differential abundance of fecal microbiota. Significant differences of fecal microbiota at the phylum level in the (**a**) Control_AF group compared to the Control_BE group, (**b**) in the TWK10_AF group compared to the TWK10_BE group and (**c**) Relative abundances of the gut microbiota at the family level among groups. Data are shown as mean ± SD. Statistical differences between before and after administration were analyzed by Wilcoxon signed-rank test. # *p* < 0.05.


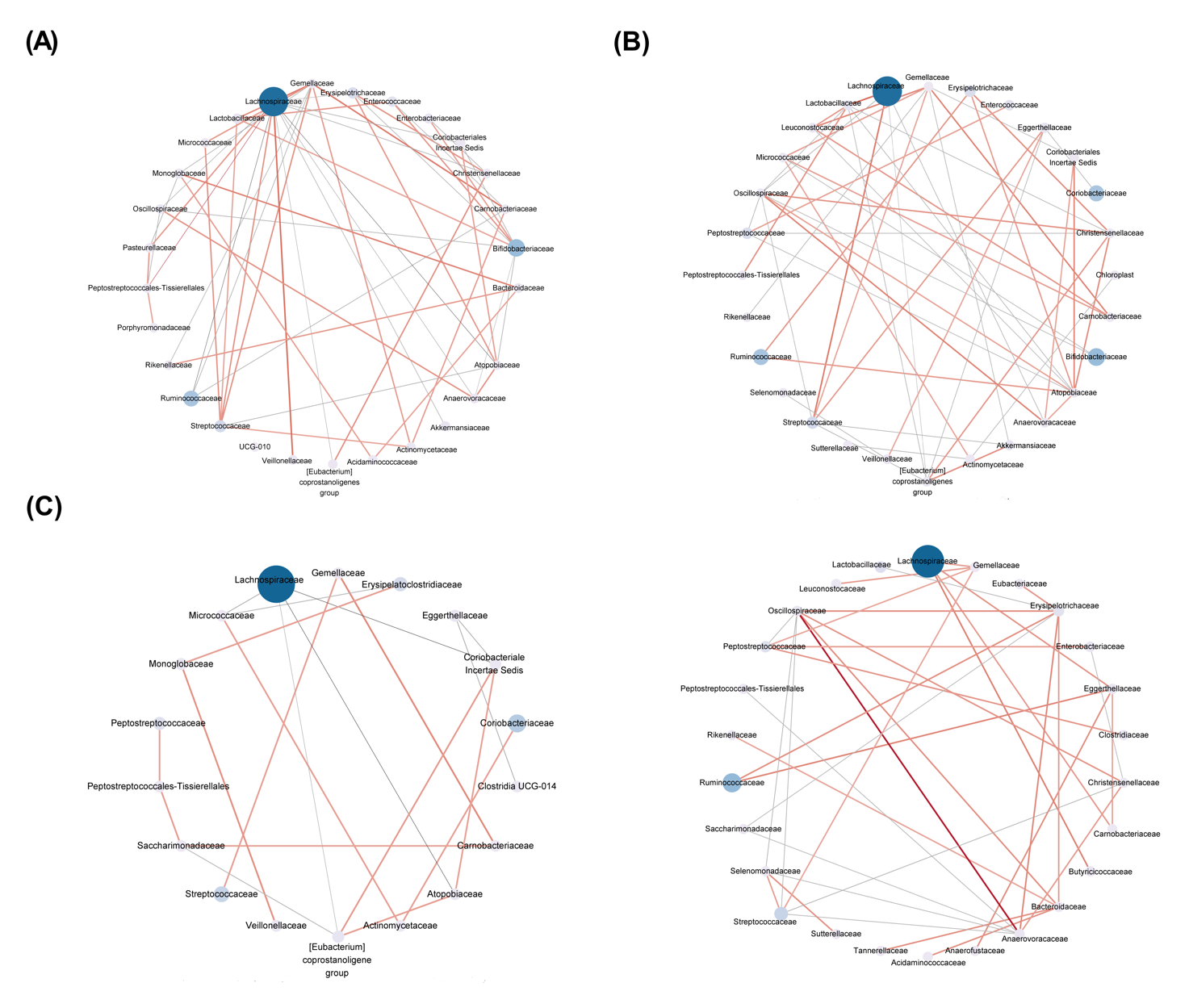


**Fig S3.** Co-occurrence network analysis of the gut microbiota. Bacterial networks were generated using SparCC correlation coefficients, based on relative abundances at the family level: (**a**) In the control group before administration (Control_BE), and correlation coefficients ranged from |0.50| to |0.97|, (**b**) In the control group after administration (Control_AF), there were 69 nodes, and correlation coefficients ranged from |0.50| to |0.82|, (**c**) In the TWK10 group before administration (TWK10_BE), and the correlation coefficients ranged from |0.51| to |0.99|and (**d**) In the TWK10 group after administration (TWK10_AF), and correlation coefficients range from |0.50| to |0.89|. Nodes represent bacteria families; grey and orange color edges represent negative and positive correlation coefficients, respectively. The size and the degree of the blue color of nodes in the network represent the relative abundance of each taxon in each group.
